# The Burden of Proof studies: assessing the evidence of risk

**DOI:** 10.1038/s41591-022-01973-2

**Published:** 2022-10-10

**Authors:** Peng Zheng, Ashkan Afshin, Stan Biryukov, Catherine Bisignano, Michael Brauer, Dana Bryazka, Katrin Burkart, Kelly M. Cercy, Leslie Cornaby, Xiaochen Dai, M. Ashworth Dirac, Kara Estep, Kairsten A. Fay, Rachel Feldman, Alize J. Ferrari, Emmanuela Gakidou, Gabriela Fernanda Gil, Max Griswold, Simon I. Hay, Jiawei He, Caleb M. S. Irvine, Nicholas J. Kassebaum, Kate E. LeGrand, Haley Lescinsky, Stephen S. Lim, Justin Lo, Erin C. Mullany, Kanyin Liane Ong, Puja C. Rao, Christian Razo, Marissa B. Reitsma, Gregory A. Roth, Damian F. Santomauro, Reed J. D. Sorensen, Vinay Srinivasan, Jeffrey D. Stanaway, Stein Emil Vollset, Theo Vos, Nelson Wang, Catherine A. Welgan, Sarah S. Wozniak, Aleksandr Y. Aravkin, Christopher J. L. Murray

**Affiliations:** 1 Institute for Health Metrics and Evaluation, University of Washington, Seattle, WA USA; 2 Department of Health Metrics Sciences, School of Medicine, University of Washington, Seattle, WA USA; 3 School of Population and Public Health, University of British Columbia, Vancouver, British Columbia Canada; 4 School of Public Health, The University of Queensland, Brisbane, Queensland Australia; 5 Queensland Centre for Mental Health Research, Wacol, Queensland Australia; 6 Department of Anesthesiology & Pain Medicine, University of Washington, Seattle, WA USA; 7 Division of Cardiology, University of Washington, Seattle, WA USA; 8 The George Institute for Global Health, The University of New South Wales, Sydney, New South Wales Australia; 9 Department of Applied Mathematics, University of Washington, Seattle, WA USA

**Keywords:** Diseases, Risk factors

## Abstract

Exposure to risks throughout life results in a wide variety of outcomes. Objectively judging the relative impact of these risks on personal and population health is fundamental to individual survival and societal prosperity. Existing mechanisms to quantify and rank the magnitude of these myriad effects and the uncertainty in their estimation are largely subjective, leaving room for interpretation that can fuel academic controversy and add to confusion when communicating risk. We present a new suite of meta-analyses—termed the Burden of Proof studies—designed specifically to help evaluate these methodological issues objectively and quantitatively. Through this data-driven approach that complements existing systems, including GRADE and Cochrane Reviews, we aim to aggregate evidence across multiple studies and enable a quantitative comparison of risk–outcome pairs. We introduce the burden of proof risk function (BPRF), which estimates the level of risk closest to the null hypothesis that is consistent with available data. Here we illustrate the BPRF methodology for the evaluation of four exemplar risk–outcome pairs: smoking and lung cancer, systolic blood pressure and ischemic heart disease, vegetable consumption and ischemic heart disease, and unprocessed red meat consumption and ischemic heart disease. The strength of evidence for each relationship is assessed by computing and summarizing the BPRF, and then translating the summary to a simple star rating. The Burden of Proof methodology provides a consistent way to understand, evaluate and summarize evidence of risk across different risk–outcome pairs, and informs risk analysis conducted as part of the Global Burden of Diseases, Injuries, and Risk Factors Study.

## Abstract (web summary)

A new Burden of Proof meta-analytic method that accounts for between-study heterogeneity and corrects for bias between different study designs is used to interpret the strength of evidence between different pairs of risk factors and health outcomes.

## Main

Exposure to different risk factors plays an important role in the likelihood of an individual developing or experiencing more severe outcomes from certain diseases, such as high blood pressure increasing the risk of heart disease or not having access to a safe water source increasing the risk of diarrheal diseases^1^. Understanding and quantifying the relationship between risk factor exposure and the risk of a subsequent outcome is therefore essential to set priorities for public policy, to guide public health practices, to help clinicians advise their patients and to inform personal health choices. Consequently, information on risk–outcome relationships can be used in the formulation of many types of public policies, including national recommendations on diet, occupational health rules, regulations on behavior such as smoking in public places, and guidance on appropriate levels of taxes and subsidies. As new evidence is continuously being produced and published, the systematic and comparable assessment of risk functions is a dynamic challenge. Up-to-date assessments of risk–outcome relationships are essential to, and a core component of, the Global Burden of Diseases, Injuries, and Risk Factors Study (GBD) comparative risk assessment (CRA)^1–3^, which aims to help decision-makers understand the magnitude of different health problems.

Evidence on risk–outcome relationships comes from many types of studies, including randomized controlled trials (RCTs), cohort studies, case-control studies, cross-sectional analyses, ecological studies and animal studies. Each study type has characteristic strengths and weaknesses. For example, RCTs are the most robust method for dealing with confounding but are often conducted with strict inclusion and exclusion criteria, meaning that trial participants are unlikely to be fully representative of the general population, as well as being done over relatively short durations^3–5^. Case-control studies are well suited for understanding the risks linked to rare outcomes but may be subject to recall bias for past exposure^6,7^. Animal studies are widely used in evaluating the risks of consumer products and environmental risks but may not be generalizable to humans^8^. Study design and analysis impacts causal interpretation and understanding of the results^9^. When synthesizing evidence from different studies, strong assumptions—usually that of a log-linear relationship between risk and exposure—are often made to increase the mathematical tractability of the analysis^10–12^. Between-study heterogeneity—that is, disagreement in study-specific inferred relationships between risk exposure and outcome—is quantified in meta-analytic summaries, and has some effect on fixed-effects variance estimates, but is not otherwise used in the overall assessments of the uncertainty in risk–outcome relationships^12,13^. Risk factors associated with comparatively modest increases in the hazard are often questioned because of the potential for residual confounding^14^. Given the very mixed evidence landscape, it is perhaps not surprising that there are so many controversies in the literature^15–18^.

While evidence is often heterogeneous, the need for clear guidance has led national advisory groups and international organizations to use expert committees to evaluate the evidence and formulate recommendations. The biggest advantage of expert groups is their ability to carefully consider nuances in the available evidence, but they are inherently subjective. For instance, expert groups across subfields of health science weight types of evidence differently, and even groups of experts within the same subfield may arrive at divergent conclusions. These expert groups often use meta-analyses of the available evidence, such as those produced by the Cochrane Collaborations^19^, as an input to their deliberations. Even Cochrane Reviews, however, allow authors to use a range of methodologies and approaches to studies on risk of bias, limiting comparability across risk–outcome pairs^19^. Tools have been produced to help standardize consideration of evidence, such as Grading of Recommendations, Assessment, Development and Evaluations (GRADE^20,21^), but while very helpful, they cannot be implemented algorithmically. No quantitative assessment of the evidence can or should substitute completely for expert deliberation, but a quantitative meta-analytic approach that addresses some of the issues identified by GRADE and others could be a useful input to international and national expert committee considerations.

Here, we propose a complementary approach, in which we quantify the mean relationship (the risk function) between risk exposure and a disease or injury outcome, after adjusting for known biases in the existing studies. Unlike existing approaches, our approach does not force log-linearity in risk functions or make additional approximations, such as midpoint approximations for ranges or shared reference groups^22–24^. To quantify the effect of bias, we considered risk of bias criteria that inform GRADE^20,21^, Cochrane Reviews^19^ and evidence-based practice, and consulted widely outside of the Institute for Health Metrics and Evaluation, including with clinicians, physicians, medical and public health researchers and national health policy-makers (for example, former Ministers of Health). We encoded these variables that are used to assess risk of bias as potential study-level bias covariates within the proposed meta-analytic framework. This approach complements GRADE and Cochrane Reviews, which require analysts to assess and flag risks of bias. We then developed the burden of proof risk function (BPRF), which complements the mean risk and is defined as the smallest level of excess risk (closest to no relationship) that is consistent with the data. To aid interpretation of the results, we classify risk–outcome pairs into five categories (star ratings of one to five) based on the average magnitude of the BPRF. To illustrate this approach to assessing risk–outcome relationships, we provide four selected examples, showing both weak and strong risk–outcome relationships.

## Results

### Overview

To support estimation of the BPRF, we developed a meta-analytic approach that addresses a number of issues that have previously limited interpretations of the available evidence. This approach relaxes the assumption that the relative risk of an outcome increases exponentially as a function of exposure, standardizes the assessment of outliers, explicitly handles the range of exposure in a study in both the ‘alternative’ groups (numerator) and ‘reference’ groups (denominator) of a relative risk, tests for systematic bias as a function of study design using automatic covariate selection, and quantifies between-study heterogeneity while adjusting for the number of studies. Using unexplained between-study heterogeneity and accounting for small numbers of studies, we estimate the BPRF as the 5th (if harmful) or 95th (if protective) quantile risk curve closest to the null (relative risk equal to 1). We flag evidence of the small-study effect (significant association between mean effect and standard error) as an indicator of potential publication or reporting bias.

We evaluated the BPRF for 180 risk–outcome pairs in the GBD CRA framework. To simplify communication, we then computed the associated risk–outcome score (ROS) for each pair by averaging the BPRF across a relevant exposure interval and converted each ROS into a star rating from one to five. One star refers to risk–outcome pairs where a conservative interpretation of the evidence—accounting for all uncertainty including between-study heterogeneity—may suggest there is no association and two–five stars refers to risk–outcome pairs where a conservative interpretation of the evidence may suggest that, for harmful effects, average exposure increases excess risk relative to the level of exposure that minimizes risk from 0 to 15% (two stars; weak evidence of association), from >15 to 50% (three stars; moderate evidence of association), from >50 to 85% (four stars; strong evidence of association) and >85% (five stars; very strong evidence of association), and for protective effects, decreases excess risk relative to no exposure from 0 to 13% (two stars), from >13 to 34% (three stars), from >34 to 46% (four stars) and >46% (five stars). The corresponding ROS thresholds for both harmful and protective risks are <0 for one star, >0–0.14 for two stars, >0.14–0.41 for three stars, >0.41–0.62 for four stars and >0.62 for five stars. Of the 180 risk–outcome pairs investigated, 40 risk–outcome pairs were given a one-star rating, 72 pairs were given a two-star rating, 46 were given a three-star rating, 14 were given a four-star rating and 8 were given a five-star rating (Table 1). Here, we present results from each step of the evaluation process for four risk–outcome pairs to demonstrate how our methodology can be applied to pairs across the ROS spectrum and across a range of available study types and risk curve shapes, varying levels of between-study heterogeneity, and varying numbers of data points and studies. These four pairs also allow us to demonstrate how policy-makers should interpret our findings for both strong and weak risk–outcome relationships.

**Table 1:** BPRF and ROS ranges associated with each star rating, and number of risk–outcome pairs assigned to each star rating

Star rating	Magnitude of BPRF range for harmful effects	Magnitude of BPRF range for protective effects	ROS range	Number of R–O pairs (*n* = 180)
One star	No association (0%)	No association (0%)	<0.000	40
Two stars	0–15%	0–13%	0.000–0.1398	72
Three stars	>15–50%	>13–34%	>0.1398–0.4055	46
Four stars	>50–85%	>34–46%	>0.4055–0.6152	14
Five stars	>85%	>46%	>0.6152	8

BPRF refers to the most conservative estimate of the magnitude of the increase in risk (for harmful risks) or decrease in risk (for protective risks) of the specified outcome with exposure to the specified risk factor. R–O, risk–outcome.

### Smoking and lung cancer (five stars)

**Fig. 1:**
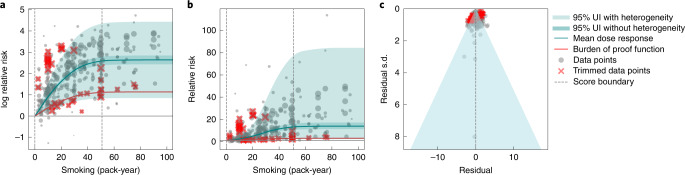
Smoking and lung cancer. **a**, Log relative risk function for smoking and lung cancer. **b**, Relative risk function for smoking and lung cancer. **c**, A modified funnel plot for smoking and lung cancer showing the residuals (relative to 0) on the *x* axis and the estimated standard deviation (s.d.) that includes reported s.d. and between-study heterogeneity on the *y* axis.

We used a standardized approach to search for and extract data from published studies on the relationship between pack-years smoked and the log relative risk of lung cancer, resulting in 371 observations from 25 prospective cohort studies and 53 case-control studies (three of them nested) reported from 1980 onwards (Fig. 1; step 1 in Methods)^25^. The studies spanned a wide range of pack-years of smoking, from nearly one to over 112 pack-years. We found the 15th percentile of exposure in the reference group to be zero pack-years (and the 85th percentile of exposure among exposed groups in the cohort studies to be 50.88 pack-years (Fig. 1a,b). On average, we found a very strong relationship between pack-years of smoking and log relative risk of lung cancer (step 2 in Methods). At 20 pack-years, the mean relative risk (an effect size measure) was 5.11 (95% uncertainty interval (UI) 1.84–14.99), and at 50.88 pack-years (85th percentile of exposure) it was 13.42 (2.63–74.59) (Fig. 1b and Supplementary Table 1). The relationship is not log-linear, with declining effects of further pack-years of smoking, particularly after 40 pack-years. In the analysis of bias covariates (step 3 in Methods), we adjusted data from studies that did not adjust for more than five confounders, including age and sex. There is enormous heterogeneity in the reported relative risk for lung cancer across studies (Fig. 1b; step 4 in Methods). In trimming 10% of observations, we identified observations both above and below the cloud of points, which we excluded (step 5 in Methods). The mixed-effects models fit the data, that is, the reported uncertainty together with estimated between-study heterogeneity covers the estimated residuals, as Fig. 1c demonstrates. Even taking the most conservative interpretation of the evidence—the 5th quantile risk function including between-study heterogeneity, or the BPRF—smoking dramatically increases the risk of lung cancer (Fig. 1a,b). There is evidence of potential reporting or publication bias (Fig. 1c). The BPRF suggests that smoking in the range of the 15th–85th percentiles of exposure raises the risk of lung cancer by an average of 106.7%, for an ROS of 0.73 (step 6 in Methods). These findings led us to classify smoking and lung cancer as a five-star risk–outcome pair.

### Systolic blood pressure and ischemic heart disease (five stars)

**Fig. 2:**
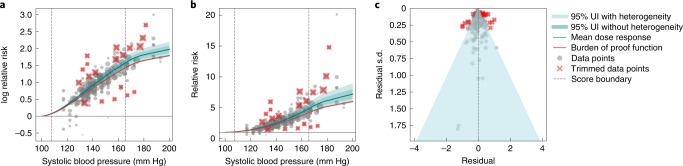
Systolic blood pressure and ischemic heart disease. **a**, Log relative risk function for systolic blood pressure and ischemic heart disease. **b**, Relative risk function for systolic blood pressure and ischemic heart disease. **c**, A modified funnel plot for systolic blood pressure and ischemic heart disease showing the residuals (relative to 0) on the *x* axis and the estimated standard deviation (s.d.) that includes reported s.d. and between-study heterogeneity on the *y* axis.

We extracted 189 observations from 41 studies (39 RCTs, 1 cohort and 1 pooled cohort) quantifying the relationship between systolic blood pressure (SBP) and ischemic heart disease (Fig. 2)^26^. We included RCTs designed to compare the health effects of different levels of blood pressure. Head-to-head trials of drug classes or combinations not designed to achieve different levels of SBP were excluded. We calculated the 15th percentile of exposure in the cohorts and trials to be an SBP of 107.5 mm Hg and the 85th percentile to be 165 mm Hg (Fig. 2a,b). The relationship is close to log-linear, although it appears to flatten out and deviate from the log-linear assumption over an SBP of 165 mm Hg (though the data are sparse over this level). An SBP of 140 mm Hg had a mean relative risk of ischemic heart disease of 2.38 (2.17–2.62) compared to 100 mm Hg, while an SBP of 165 mm Hg had a mean relative risk of 4.48 (3.81–5.26) compared to 100 mm Hg. (Fig. 2b and Supplementary Table 2). Trimming removed 10% of outlying observations with high relative risk at SBP levels between 125 and 180 mm Hg and low relative risk at SBP levels between 130 and 175 mm Hg (Fig. 2b). In the analysis of bias covariates, we found that none had a significant effect. Because the RCTs and cohorts are very consistent and because there are many consistent studies within each type, between-study heterogeneity is small (Fig. 2a,b). While there is little asymmetry in the funnel plot (Fig. 2c), we found statistically significant evidence of small-study bias using an Egger’s regression (Egger’s regression *P* value <0.05). Given the small between-study heterogeneity, the BPRF suggests that SBP in the range from the 15th to 85th percentile of exposure raises the risk of ischemic heart disease by an average of 101.36%, for an ROS of 0.70. These findings led us to classify SBP and ischemic heart disease as a five-star risk–outcome pair.

### Vegetable consumption and ischemic heart disease (two stars)

**Fig. 3:**
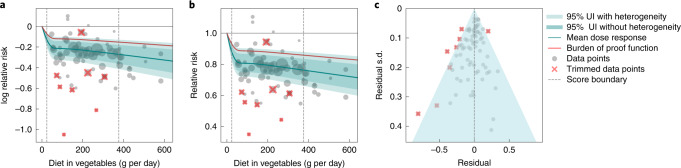
Vegetable consumption and ischemic heart disease. **a**, Log relative risk function for vegetable consumption and ischemic heart disease. **b**, Relative risk function for vegetable consumption and ischemic heart disease. **c**, A modified funnel plot for vegetable consumption and ischemic heart disease showing the residuals (relative to 0) on the *x* axis and the estimated standard deviation (s.d.) that includes reported s.d. and between-study heterogeneity on the *y* axis.

Figure 3 summarizes the cohort data on vegetable consumption and ischemic heart disease using 78 observations from 17 cohort studies^27^. The relationship is not log-linear. We found that on average, vegetable consumption was protective, with the relative risk of ischemic heart disease being 0.81 (0.74–0.89) at 100 grams per day vegetable consumption compared to 0 grams per day (Supplementary Table 3). Incrementally higher levels of exposure are associated with less steep declines in relative risk compared to those at lower levels of exposure (Fig. 3b). For this pair, trimming removed one observation that suggested a weaker protective effect size than the mean estimate, and seven observations that suggested a stronger protective effect than the mean estimate. Including between-study heterogeneity expanded the UI only slightly (Fig. 3a,b), suggesting strong agreement between studies. In the analysis of bias covariates, three were found to have a significant effect: incomplete confounder adjustment, incidence outcomes only and mortality outcomes only. The funnel plot (Fig. 3c) shows that after trimming, residual standard error (reflecting both study data variance and between-study heterogeneity) is within the expected range of the model. While there is little asymmetry in the funnel plot (Fig. 3c), we found statistically significant evidence of small-study bias using an Egger’s regression (Egger’s regression *P* value = 0.044). The BPRF suggests that vegetable consumption in the range of the 15th to the 85th percentile lowers risk of ischemic heart disease by 12.10% on average (ROS of 0.13). This leads to vegetable consumption and ischemic heart disease being classified as a two-star pair.

### Unprocessed red meat and ischemic heart disease (two stars)

**Fig. 4:**
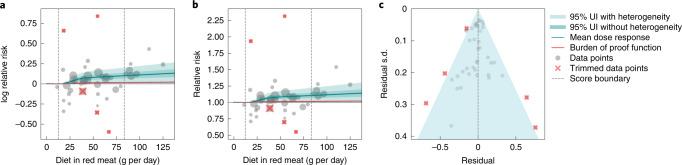
Unprocessed red meat consumption and ischemic heart disease. **a**, Log relative risk function for unprocessed red meat consumption and ischemic heart disease. **b**, Relative risk function for unprocessed red meat consumption and ischemic heart disease. **c**, A modified funnel plot for unprocessed red meat consumption and ischemic heart disease showing the residuals (relative to 0) on the *x* axis and the estimated standard deviation (s.d.) that includes reported s.d. and between-study heterogeneity on the *y* axis.

We identified 43 observations from 11 prospective cohort studies on unprocessed red meat and ischemic heart disease (Fig. 4)^28^. At an exposure of 50 grams per day, the mean relative risk is 1.09 (0.99–1.18) compared to 0 grams per day, and at 100 grams per day, it is 1.12 (0.99–1.25) (Fig. 4b and Supplementary Table 4). In the analysis of bias covariates, we found that none had a significant effect. Trimming removed five observations that reported extreme values across the range of red meat consumption. There is no visual evidence or finding of potential publication or reporting bias (Fig. 4c).

For unprocessed red meat and ischemic heart disease, the exposure-averaged BPRF is 0.01, essentially on the null threshold (Fig. 4a), equating to an ROS of 0.01, with a corresponding increase in risk of 1.04%. These findings led this risk–outcome pair to be classified as a (nominal) two stars, on the threshold between weak evidence and no evidence of association for the risk–outcome pair.

### Model validation

**Fig. 5:**
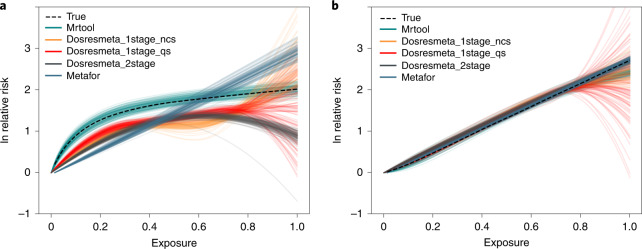
Model validation for the data-rich scenario. **a**, Estimated risk curves across 100 realizations for all methods for non-log linear risks. **b**, Estimated risk curves across 100 realizations for all methods for log linear risks. Mrtool is the method used in this paper. Dosresmeta_1stage_ncs refers to the Dosresmeta package with a natural cubic spline, while Dosresmeta_1stage_qs refers to the same tool with a quadratic spline. Dosresmeta_2stage refers to the the 2 stage approach in Dosresmeta. Metafor refers to a standard package that assumes a log linear relationship. (See ’Model validation’ for more details).

**Extended Data Fig. 1:**
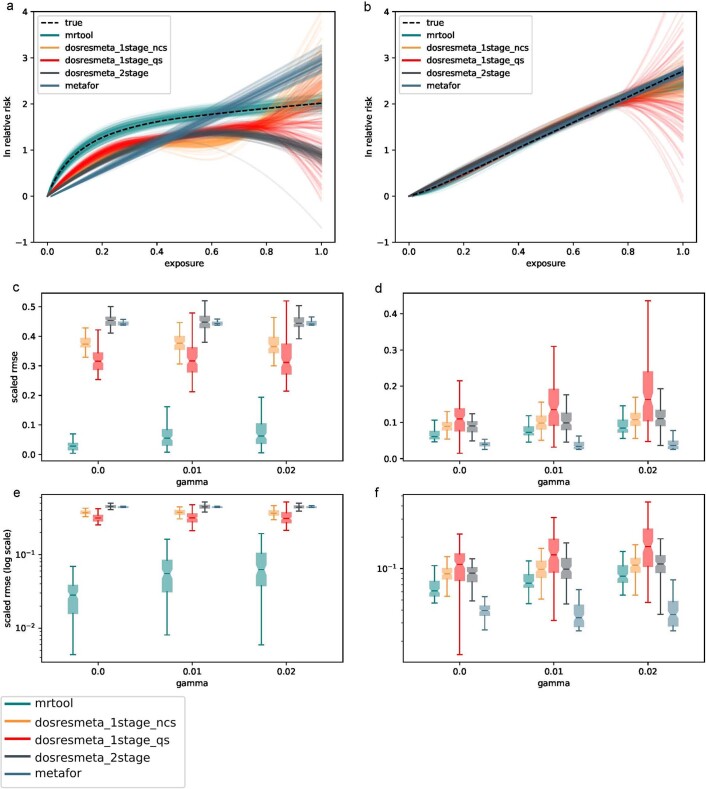
Scenario 1 mean risk curve. **a**, estimated risk curves across 100 realizations for all methods, non-log linear. **b**, estimated risk curves across 100 realizations for all methods, log linear. **c**, RMSE results summarizing the errors of each method against simulated ground truth across different levels of true between-study heterogeneity, non-log linear risks. **d**, RMSE results summarizing the errors of each method against simulated ground truth across different levels of true between-study heterogeneity, log linear risks. **e**, log-scale RMSE results summarizing the errors of each method against simulated ground truth across different levels of true between-study heterogeneity, non-log linear risks. **f**, log-scale RMSE results summarizing the errors of each method against simulated ground truth across different levels of true between-study heterogeneity, log linear risks. MRSE = root mean squared error.

**Extended Data Fig. 2:**
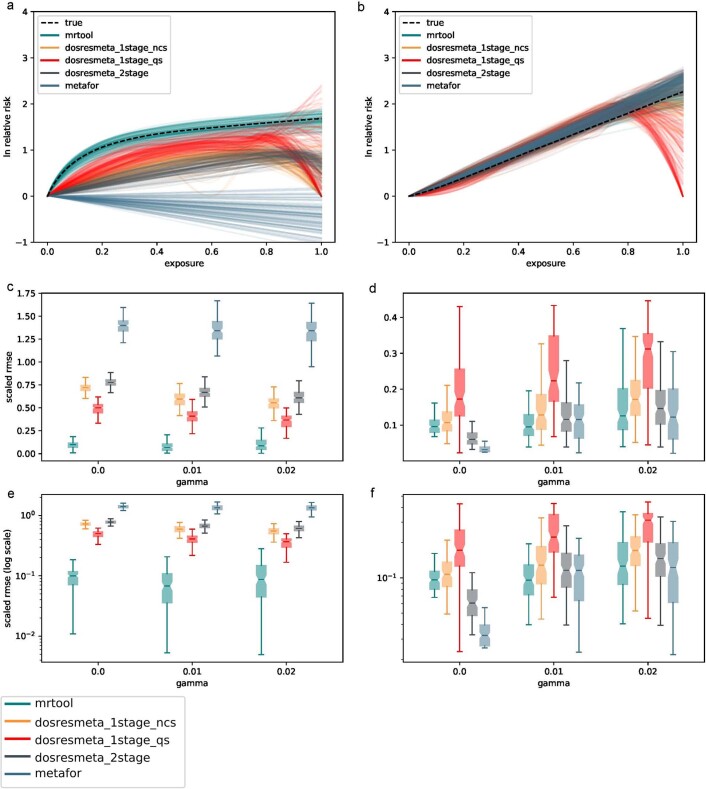
Scenario 1 burden of proof risk function. **a**, estimated risk curves across 100 realizations for all methods, non-log linear. **b**, estimated risk curves across 100 realizations for all methods, log linear. **c**, RMSE results summarizing the errors of each method against simulated ground truth across different levels of true between-study heterogeneity, non-log linear risks. **d**, RMSE results summarizing the errors of each method against simulated ground truth across different levels of true between-study heterogeneity, log linear risks. **e**, log-scale RMSE results summarizing the errors of each method against simulated ground truth across different levels of true between-study heterogeneity, non-log linear risks. **f**, log-scale RMSE results summarizing the errors of each method against simulated ground truth across different levels of true between-study heterogeneity, log linear risks. MRSE = root mean squared error.

**Extended Data Fig. 3:**
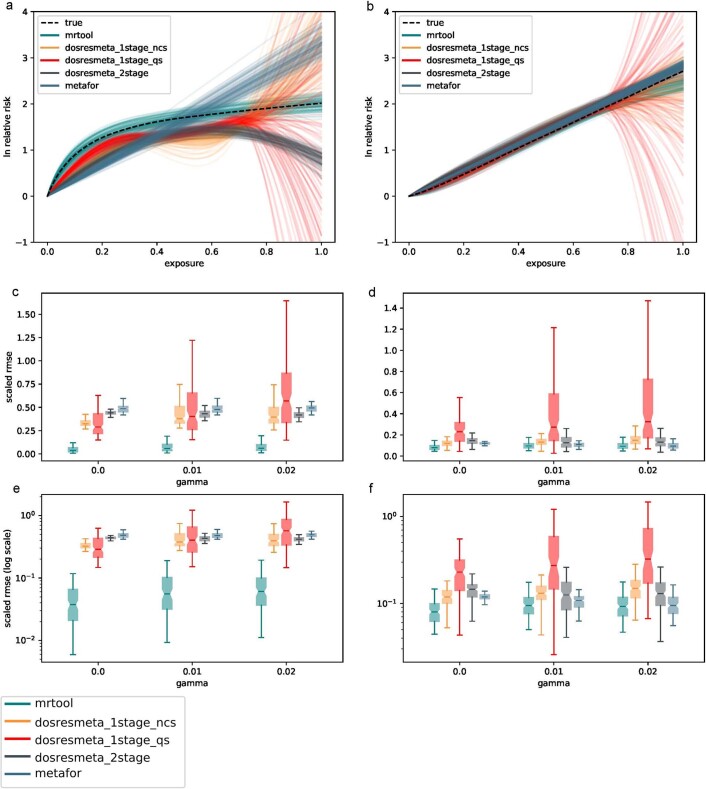
Scenario 2 mean risk curve. **a**, estimated risk curves across 100 realizations for all methods, non-log linear. **b**, estimated risk curves across 100 realizations for all methods, log linear. **c**, RMSE results summarizing the errors of each method against simulated ground truth across different levels of true between-study heterogeneity, non-log linear risks. **d**, RMSE results summarizing the errors of each method against simulated ground truth across different levels of true between-study heterogeneity, log linear risks. **e**, log-scale RMSE results summarizing the errors of each method against simulated ground truth across different levels of true between-study heterogeneity, non-log linear risks. **f**, log-scale RMSE results summarizing the errors of each method against simulated ground truth across different levels of true between-study heterogeneity, log linear risks. MRSE = root mean squared error.

**Extended Data Fig. 4:**
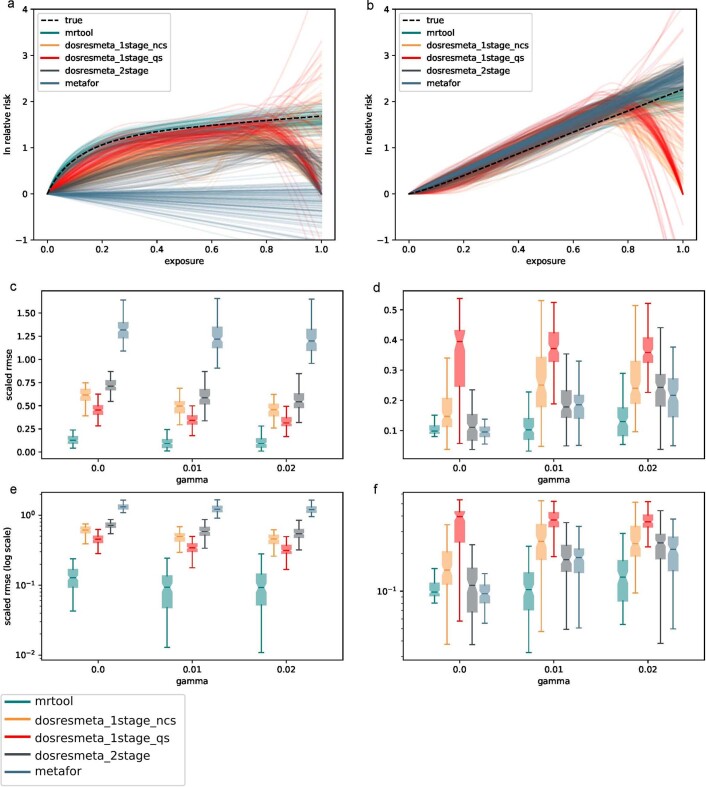
Scenario 2 burden of proof risk function. **a**, estimated risk curves across 100 realizations for all methods, non-log linear. **b**, estimated risk curves across 100 realizations for all methods, log linear. **c**, RMSE results summarizing the errors of each method against simulated ground truth across different levels of true between-study heterogeneity, non-log linear risks. **d**, RMSE results summarizing the errors of each method against simulated ground truth across different levels of true between-study heterogeneity, log linear risks. **e**, log-scale RMSE results summarizing the errors of each method against simulated ground truth across different levels of true between-study heterogeneity, non-log linear risks. **f**, log-scale RMSE results summarizing the errors of each method against simulated ground truth across different levels of true between-study heterogeneity, log linear risks. MRSE = root mean squared error.

**Extended Data Fig. 5:**
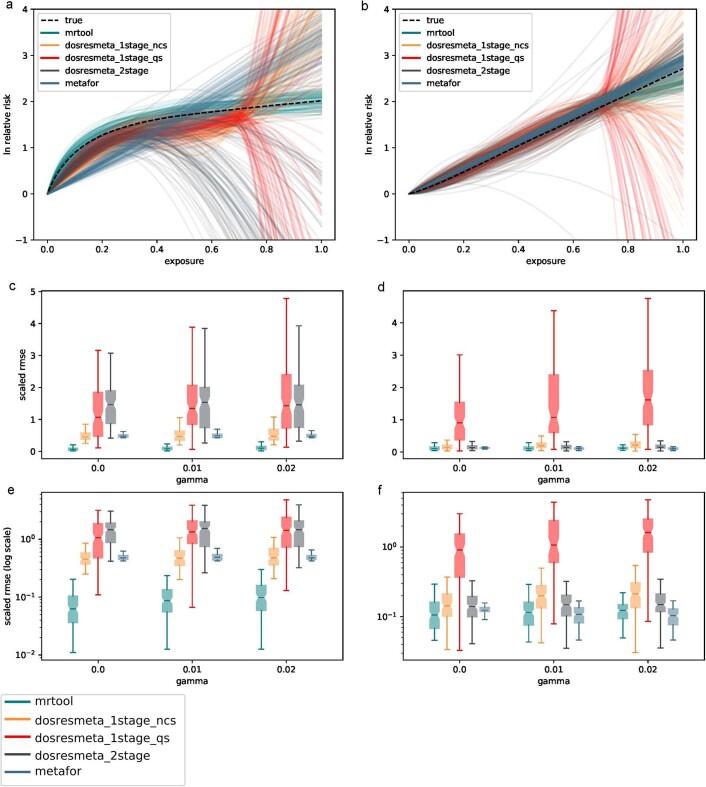
Scenario 3 mean risk curve. **a**, estimated risk curves across 100 realizations for all methods, non-log linear. **b**, estimated risk curves across 100 realizations for all methods, log linear. **c**, RMSE results summarizing the errors of each method against simulated ground truth across different levels of true between-study heterogeneity, non-log linear risks. **d**, RMSE results summarizing the errors of each method against simulated ground truth across different levels of true between-study heterogeneity, log linear risks. **e**, log-scale RMSE results summarizing the errors of each method against simulated ground truth across different levels of true between-study heterogeneity, non-log linear risks. **f**, log-scale RMSE results summarizing the errors of each method against simulated ground truth across different levels of true between-study heterogeneity, log linear risks. MRSE = root mean squared error.

**Extended Data Fig. 6:**
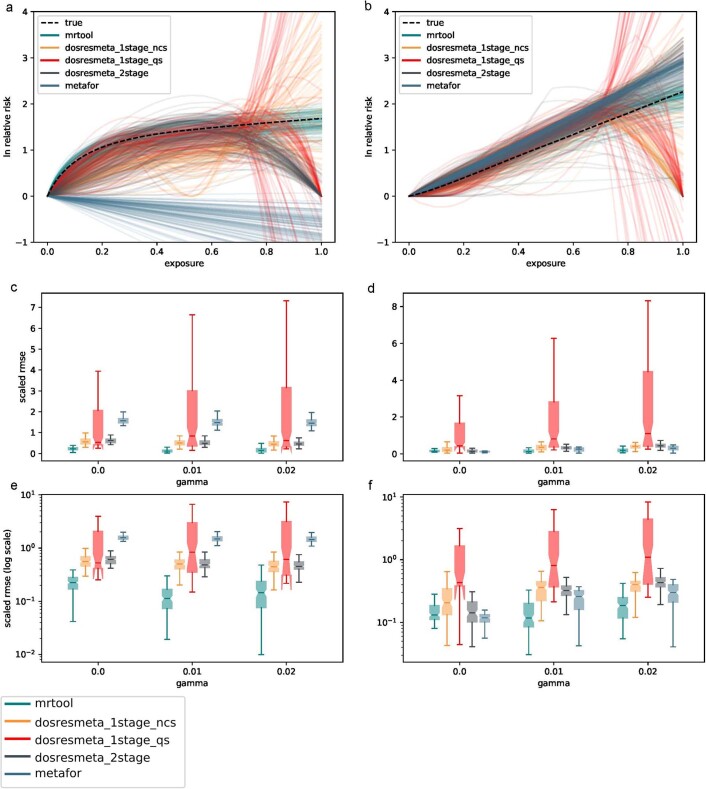
Scenario 3 burden of proof risk function. **a**, estimated risk curves across 100 realizations for all methods, non-log linear. **b**, estimated risk curves across 100 realizations for all methods, log linear. **c**, RMSE results summarizing the errors of each method against simulated ground truth across different levels of true between-study heterogeneity, non-log linear risks. **d**, RMSE results summarizing the errors of each method against simulated ground truth across different levels of true between-study heterogeneity, log linear risks. **e**, log-scale RMSE results summarizing the errors of each method against simulated ground truth across different levels of true between-study heterogeneity, non-log linear risks. **f**, log-scale RMSE results summarizing the errors of each method against simulated ground truth across different levels of true between-study heterogeneity, log linear risks. MRSE = root mean squared error.

To validate key aspects of the meta-regression tool, we ran detailed simulation experiments (step 7 in Methods). We found that the approach proposed in this study outperformed existing approaches, particularly for non-log-linear relationships (Fig. 5 and Extended Data Figs. 1–6).

## Discussion

Using a meta-analytic approach built using open-source tools, we estimated both the mean risk function and the BPRF for 180 risk–outcome pairs and assigned them a star rating based on the strength of the evidence (indicated by ROS that aggregate BPRF across standard exposure ranges) and severity of the risk. We achieved this by capturing the shape of the relationship between exposure and the risk of an outcome, detecting outliers using robust statistical methodology (trimming), testing and correcting for bias related to study design, and estimating between-study heterogeneity, adjusted for the number of studies. The BPRF is the level of elevated risk for a harmful factor (or the level of reduced risk for a protective factor) based on the most conservative (closest to null) interpretation compatible with the available evidence. It is a reflection of both the magnitude of the risk and the extent of the uncertainty surrounding the mean risk function. The four examples in the results section demonstrate the range of evidence, between-study heterogeneity and mean relative risks across risk–outcome pairs, and how these factors impact the BPRF and star rating. Importantly, only 22 of 180 pairs received a four- or five-star rating (12.22%), whereas 112 received a one- or two-star rating (62.22%).

The BPRF and associated star ratings, as well as the background rates of burden for the outcomes of concern, are intended to be useful for informing individual choices on risk exposure. For example, harmful risk–outcome pairs with four- and five-star ratings are associated with an increase in risk of more than 50% for the exposed (and more than a 34% decrease in risk for protective risks), even based on the most conservative interpretation of the evidence. For these risks, the mean effect size is often much higher. Harmful risk–outcome pairs with three stars have average increases in risk ranging from more than 15% to 50% (and a decrease of at least 13–34% for protective risks), even in the BPRF, and may be much higher depending on the individual level of risk exposure. Further, some risks have high star ratings for multiple outcomes, such as high systolic blood pressure increasing risk of ischemic heart disease and stroke, and smoking increasing risk of lung cancer, aortic aneurysm, peripheral artery disease, laryngeal cancer and other pharynx cancer (all five-star pairs), which should be considered when making individual decisions around risk exposure. Conversely, individuals can reasonably pay less attention to risks with a one-star rating. These may be real risks with small but meaningful benefits for individuals if their exposure is reduced, but the existing evidence is too limited to make stronger conclusions. Of course, individual choice should also be informed by the background risk of an outcome for an individual and the totality of risk–outcome pairs associated with a risk; a five-star relationship for a rare outcome may not be something that an individual would choose to act on, whereas three-star ratings for one risk and a set of common outcomes may warrant more action.

While the general public and committees formulating guidelines on individual behaviors—such as recommended diets—should pay attention to the star ratings, policy-makers should consider the impact of all risk–outcome pairs, not only those with high star ratings. These higher-star relationships should reassure decision-makers that the evidence supporting a risk factor is strong, but it would be unwise for decision-makers to ignore all one- and two-star risk–outcome pairs. The precautionary principle implies that public policy should pay attention to all potential risks. Lower star rating risk–outcome pairs may turn out to be null as evidence accumulates, but it is unlikely that a set of one-star risks will all turn out to be null. Public policy to address risks, even those where the BPRF indicates that risk is small or even nonexistent, will, on average, improve health. At the same time, investing in more widespread data collection for pairs with lower star ratings will reduce uncertainty and allow policy-makers to be more strategic in addressing potential risks (as star ratings may go up or down with more evidence). For example, due to very high heterogeneity between studies, a conservative interpretation of the available evidence suggests that there is weak to no evidence of an association between red meat consumption and ischemic heart disease. There is, therefore, a critical need for more large-scale, high-quality studies on red meat consumption so policy-makers can make better-informed decisions about how to prioritize policies that address this potential risk. Moreover, public policy should pay attention not only to the risk functions that are supported by evidence but the prevalence of exposure to those risks. For example, a two-star risk with high prevalence of exposure could pose a greater risk at the population level than a five-star risk with low prevalence of exposure. The GBD CRA^1–3^ provides a framework for incorporating the BPRF, the prevalence of exposure and the background rates of specific outcomes to help policy-makers evaluate the importance of risk–outcome pairs across the full range of star ratings. In the future, risk–outcome pairs with one- and two-star ratings should be investigated further through more robust, well-powered research, especially for those risks where exposure and outcome are common, so policy-makers and individuals alike can better understand whether there is a real association between risk and outcome.

The BPRF and associated star ratings have immediate applications for GBD and its users. For GBD 2020, 180 risk–outcome pairs have so far been analyzed using this approach. The remaining risk–outcome pairs will be evaluated using this meta-analytic approach in subsequent GBD rounds. Since different users will be interested in the GBD results focusing on certain star rating categories, we have developed online visualization tools (https://vizhub.healthdata.org/burden-of-proof/) that allow users to filter results by star rating. Providing dynamic tools with this capability will empower users with different thresholds for considering risk–outcome pairs and will allow broader audiences to access this information. These tools are intended to fill in a gap in the landscape of risk assessment accessibility and transparency.

**Extended Data Fig. 7:**
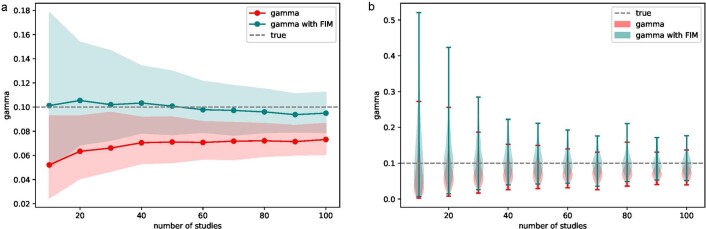
Simulation study for γ estimation as a function of the number of studies, using 500 realizations of each setting. **a**, 5th and 95th percentiles of the estimated γ values. **b**, violin plots to illustrate how the number of studies affects γ estimation. In both panels, it is clear that using FIM improves the quality of γ estimation. FIM = Fischer information matrix. γ = between-study heterogeneity parameter.

The standard approach to estimate the relationship between a risk and outcome has been to compute the mean across the universe of studies. We believe, however, that it is useful to report both the mean risk function and the BPRF, and that the more conservative interpretation may be more appropriate, particularly for exposures associated with small increases in risk, because of the risk of residual confounding. By including between-study heterogeneity in the uncertainty estimation and using this estimated uncertainty to compute a 5th or 95th quantile risk function (our BPRF), our risk assessment accounts for results that vary drastically across studies even after correcting for biases due to study design. This highlights the importance of accounting for unexplained between-study heterogeneity when estimating uncertainty and significance testing. In particular, when the BPRF spans zero (that is, the risk is one star), a conservative interpretation of the evidence is consistent with no association between the risk and the outcome. We argue that the field should eventually move to incorporating between-study heterogeneity into significance testing of the mean function. Our meta-analytic approach uses splines to estimate the shape of the risk function without imposing a functional form such as log-linearity, and can be widely applied to other risk–outcome pairs not included in this analysis. This flexibility is an important strength of our approach because many risk–outcome pairs do not have a log-linear relationship. When there are strong threshold effects, log-linear risk functions can exaggerate risk at higher exposure levels and obfuscate important detail at lower exposure levels. This more flexible approach helps identify the true shape of the risk function. Previously, the main challenge had been that if the assumption of log-linearity is relaxed, the level of risk exposure matters, so comparisons between an exposed group and a reference group needed to take into account the range of exposure in each group. We dealt with this problem directly by integrating the risk function over a range of exposures and including this mechanism in the likelihood. Our approach may be of use for meta-analyses in many areas, even if the analyst is only interested in the mean function. The model validation analysis (step 7 in Methods; Extended Data Figs. 1–7) demonstrates that our approach captures non-log-linear functions with significantly greater accuracy compared to existing dose–response meta-regression tools while still capturing log-linear relationships when present. The advantage in accuracy over existing tools increases in data-sparse cases that have fewer studies and observations. Our approach is also robust to bias covariates, such as study type, since we explicitly test for the impact of these covariates using a Lasso framework, and then adjust the estimated risk curves using covariates that are found to be statistically significant.

**Extended Data Fig. 8:**
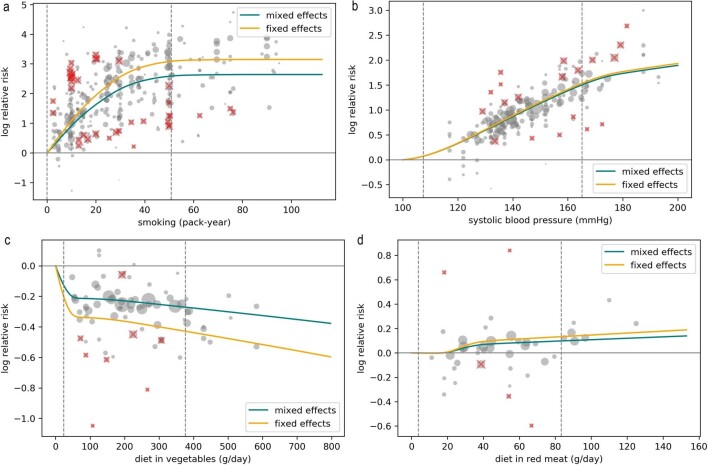
Sensitivity study results comparing mixed effects model (teal) and fixed effects model (orange). **a**, smoking and lung cancer. **b**, systolic blood pressure and ischemic heart disease. **c**, vegetables and ischemic heart disease. **d**, unprocessed red meat and ischemic heart disease.

**Extended Data Fig. 9:**
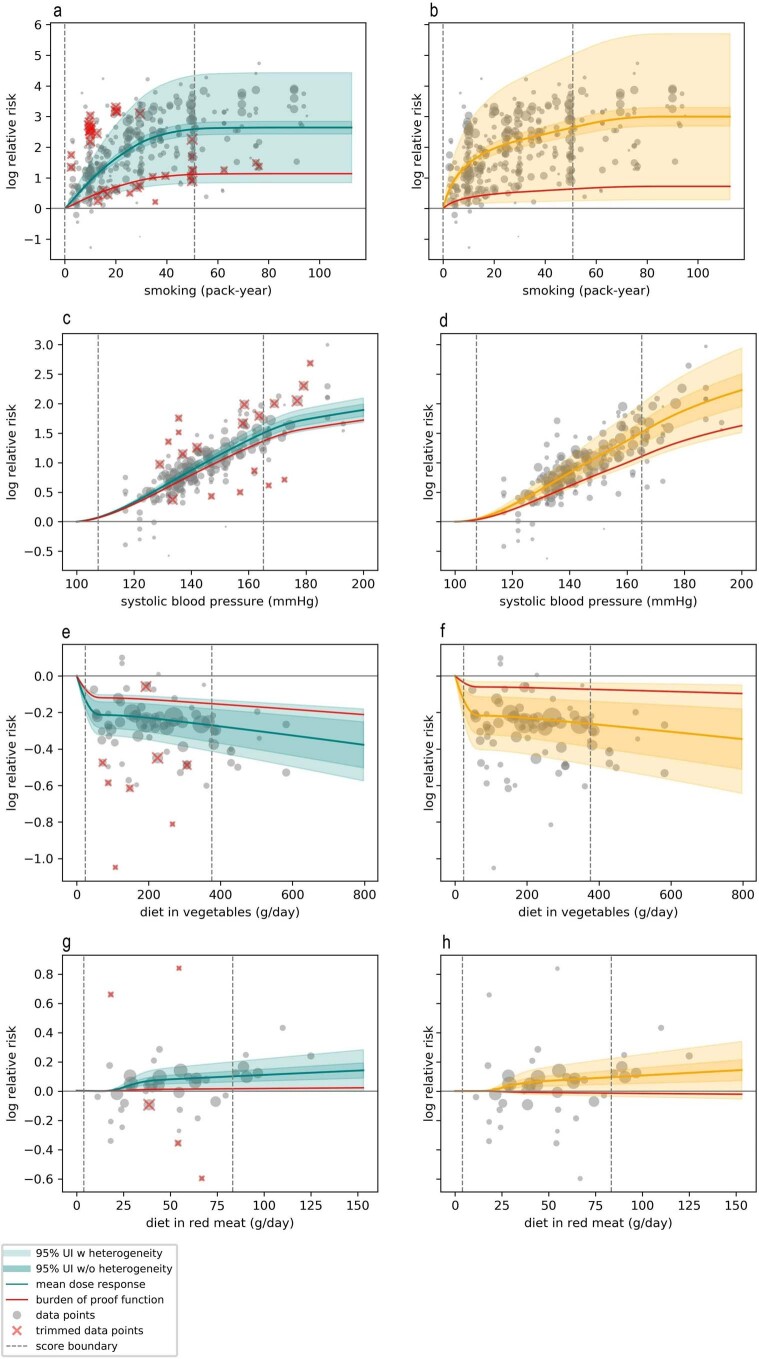
Sensitivity study results comparing trimming (teal) and no trimming (orange). **a**, smoking and lung cancer, with trimming. **b**, smoking and lung cancer, no trimming**. c**, systolic blood pressure and ischemic heart disease, with trimming. **d**, systolic blood pressure and ischemic heart disease, no trimming. **e**, vegetables and ischemic heart disease, with trimming. **f**, vegetables and ischemic heart disease, no trimming. **g**, unprocessed red meat and ischemic heart disease, with trimming. **h**, unprocessed red meat and ischemic heart disease, no trimming.

The proposed framework uses robust methodology (trimming) to make the approach robust to outlying observations. Sensitivity analyses (step 8 in Methods; Extended Data Figs. 8, 9 and Supplementary Table 5) show that trimming 10% of the data makes estimation more stable and reliable. Trimming automatically focuses on unexplained large errors, and in practice does not remove ‘gold standard’ data points.

The intention of this approach to evaluating risk–outcome associations is to complement the existing GRADE and Cochrane Review frameworks for assessing evidence and making recommendations. Our suite of meta-analyses address many of the limitations of GRADE and Cochrane by focusing on study design factors whose impact can be assessed quantitatively from the body of studies informing the analysis rather than requiring analysts to assess and flag risks of bias. Thus, we believe our approach contributes important information for expert deliberation.

This framework has a number of limitations. First, there are qualitative considerations about study design and execution that may be hard to capture in a set of structured risk of bias covariates. Our framework for adjusting for study design is necessarily limited to observable study design characteristics. Further, while our approach offers a rigorous way to combine results from different studies and different types of studies, fundamentally discordant evidence or types of evidence, such as chemical experiments, do not lend themselves to direct inclusion in this framework. Second, the trimming approach requires a user-specified level of outliers, and although fitting 90% of the data works well in practice, automated techniques to estimate the potential level of outliers in the dataset would strengthen the approach. Third, while our approach can explicitly test for potential publication or reporting bias related to the association of reported effect sizes and standard errors, other types of publication bias are more challenging to evaluate, namely when studies are more consistent with each other than expected by chance. In these cases, the Fisher information matrix approach is still helpful, particularly when the number of studies is small, because it is guaranteed to provide a quantile of heterogeneity even if the heterogeneity parameter is estimated to be 0. Fourth, our study bias covariates cannot fully capture and correct for bias if all or even the vast majority of the input studies are biased. Fifth, including pooling studies in the meta-regression, though extremely useful in providing robust estimates of mean effects, may artificially decrease our estimates of between-study heterogeneity because many of these studies do not publish measures of between-study heterogeneity across cohorts. We partially addressed this issue by using the Fisher information matrix approach to estimate the plausible between-study heterogeneity, but more emphasis on between-study heterogeneity in future pooling studies would allow users to better interpret the generalizability of the mean effects. Sixth, to avoid overfitting bias covariates, we used Gaussian priors on the bias covariates so that these relationships were only detected when there were sufficient studies supporting the estimate of the bias. Alternative priors could increase or decrease the biases that are detected. Seventh, we estimated the BPRF and translated this into a star rating for each risk–outcome pair. There may be ranges of exposure within which there is a marked increase in the BPRF, but over most of the range, the increase in risk is small. Giving different star ratings to different ranges of exposure would, however, add a further degree of complexity that we sought to avoid. Eighth, we had no direct way of introducing or including animal studies and are thus agnostic to that evidence category.

We developed a data-driven meta-analytic approach, using open-source computational tools cited in this study, that identifies the shape of the risk–outcome relationship and robustly quantifies between-study heterogeneity after correcting for bias correlated with attributes of study design. We used this risk function to estimate both the mean relationship and BPRF for 180 risk–outcome pairs. The BPRF provides the most conservative interpretation of the severity of risk based on the available evidence. Using the BPRF, we classified risk–outcome relationships into five categories based on the strength of the Burden of Proof relationship. This standardized tool cannot address every nuance in the interpretation of the available data but can quantify a wide range of dimensions previously addressed in more subjective and qualitative ways, particularly in conjunction with information on risk exposure prevalence and outcome burden. We intend to update these risk functions over time so that they reflect the latest available evidence, including adding new risk–outcome relationships as new evidence is published. The BPRF and associated star ratings improve the field of comparative risk assessment and increase the transparency of expert deliberation of human health risks. The star ratings can be used to assess risk and inform individual and policy-level decisions around risk exposure prevention, public health guidance and personal health choices.

## Methods

### Overview

Our meta-analytic approach followed six main steps: (1) search and extract data from published studies using a standardized approach; (2) estimate the shape of the exposure versus relative risk relationship, integrating over the exposure ranges in different comparison groups and avoiding the distorting effect of outliers; (3) test and adjust for systematic biases as a function of study attributes; (4) quantify remaining between-study heterogeneity while adjusting for within-study correlation induced by computing the relative risks for several alternatives with the same reference, as well as the number of studies; (5) assess evidence for small-study effects to evaluate a potential risk of publication or reporting bias; and (6) estimate the BPRF, quantifying a conservative interpretation of the average risk increase across the range of exposure supported by the evidence to compute the ROS, and map the ROS into five categories of risk. Zheng and colleagues^29^ published the technical developments required to implement this approach, which are also disseminated using open-source Python libraries^30,31^. We validated the model through simulation studies, and then applied our meta-analytic approach to 180 risk–outcome pairs. We present our findings for four pairs that demonstrate a range of risk relationships (smoking and lung cancer, systolic blood pressure and ischemic heart disease, vegetable consumption and ischemic heart disease, and red meat consumption and ischemic heart disease). This study complies with the Guidelines on Accurate and Transparent Health Estimate Reporting recommendations (Supplementary Table 6)^32^.

### Searching for and extracting published data following a standardized protocol

For each risk–outcome pair, we used standard search strings or Preferred Reporting Items for Systematic Reviews and Meta-Analyses (PRISMA) guidelines to identify data from research databases and other sources. Data were then extracted from studies that met inclusion criteria using uniform extraction procedures. We mapped all outcomes in the data to GBD cause categories to produce a standard set of outcomes. When necessary, this process included mapping a standard set of other, often narrower, outcomes to GBD causes. When the lower end of exposure levels was not reported, we used the 15th percentile of known exposure in microdata sources; when the upper end was not reported, we used the width of the adjacent exposure interval; and when neither end was reported, we used the GBD global exposure distribution. See Supplementary Tables 7–10 for detailed information on the included data sources for each example risk–outcome pair. For a full description of how each of the four selected risk–outcome pairs were defined and measured, how data inputs were identified and assessed for eligibility, the summary results from input studies and other information on conducting systematic reviews for each risk, see Dai et al.^25^, Razo et al.^26^, Stanaway et al.^27^, and Lescinsky et al.^28^. Detailed PRISMA checklists are also included in the supplementary information of each of these articles.

### Estimating the shape of the risk–outcome relationship

Most classic epidemiological analyses of dose–response risk relationships have either assumed the relationship between risk and outcome to be log-linear or converted continuous exposure variables into dichotomous exposure categories. This assumption simplifies the analysis considerably. Unfortunately, although assuming a log-linear relationship is analytically convenient and allows for the use of simple open-source tools^10^, it is not necessarily biologically or clinically plausible (see step 7 in Methods for more details). For some risks, such as smoking, the log relative risk of the outcome flattens at higher exposures. For others, the log relative risk curves are J-shaped. We therefore chose to estimate the shape of the relationship directly from the data using a regularized spline.

Nonlinear modeling of dose–response relationships brings new challenges that are not present in the log-linear case. Rather than simply using midpoints of the data, we need to account for interval exposures in reference and alternative exposure groups. Moreover, the observation mechanism that accounts for this level of detail is nonlinear. Model stability becomes an important problem to make the approach systematically applicable across a broad range of cases. Accounting for outliers in the data becomes more important. Finally, capturing between-study heterogeneity in a tractable and stable way is important. Our approach is sensitive to the level of and difference in exposure, and explicitly handles the lack of common reference groups and exposure ranges for reference and alternative groups that are present in a vast majority of risk–outcome analyses. The statistical approach includes four aspects that make it useful for estimating risk–outcome relationships.

#### Basis splines, measurement mechanism and shape constraints

We used a Bayesian regularized spline to obtain the general shape of the nonlinear relationship. Basis splines represent nonlinear curves as linear combination of recursively generated basis elements^33^. The basis elements were recursively generated using piecewise smooth polynomials, and were roughly localized to certain regions of the exposure variable in the data. Most of the time, quadratic or cubic polynomials were used, often with linear tails in the presence of sparse data. This approach allowed the common restricted cubic spline and constraints on the shape of the relationship (including nondecreasing and nonincreasing).

Given basis functions *f*_1_, …, *f*_*k*_ and coefficient vector *β* = (*β*_1_,..., *β*_*k*_), the final curve is obtained as a *β*-linear combination

$${\rm{signal}} = \beta _1f_1 + \cdots + \beta _kf_k.$$

Specifically, for any given exposure (*x*), the prediction using the spline model is given by

1 $${\mathrm{signal}}\left( x \right) = \beta _1f_1\left( x \right) + \cdots + \cdots + \beta _kf_k\left( x \right) = \left\langle {\mathbf{{X}},\beta } \right\rangle$$

where **X** is a vector containing (*f*_1_(*x*), …, *f*_*k*_(*x*)). Derivatives and integrals of splines can likewise be expressed as linear combinations of spline coefficient *β*. For additional details about *B*-splines see Zheng et al.^29^

Many studies of dose–response relationships report relative risks between categories defined by intervals of consumption. In mathematical notation, these observations are given by

2 $$y_{ij} = \frac{{\frac{1}{{d_{ij} - c_{ij}}} {\smallint }_{c_{ij}}^{d_{ij}} f\left( x \right){\mathrm{d}}x}}{{\frac{1}{{b_{ij} - a_{ij}}} {\smallint }_{a_{ij}}^{b_{ij}} f\left( x \right){\mathrm{d}}x}},$$

where *y*_*ij*_ is the reported relative risk corresponding to measurement *j* in study *i*, [*a*_*ij*_,*b*_*ij*_] delineates the reference group exposure interval, and [*c*_*ij*_,*d*_*ij*_] delineates the alternative group exposure interval.

When *f*(*x*) is represented using a spline, each integral is a linear function of *β* similar to equation (1). The model (equation (2)) is then a ratio of linear functions,

3 $$y_{ij} = f_{ij}\left( \beta \right): = \frac{{\left\langle {X_{ij}^1,\beta } \right\rangle }}{{X_{ij}^2,\beta }}.$$

with the log relative risk given by

4 $$\ln \left( {y_{ij}} \right) = \ln \left( {\left\langle {X_{ij}^1,\beta } \right\rangle } \right) - \ln\left( {\left\langle {X_{ij}^2,\beta } \right\rangle } \right).$$

Equation (4) is a nonlinear function of the spline coefficients *β*.

When studying dose–response relationships, we allow for shape constraints of the inferred mean response. For example, for some harmful risks, such as smoking and air pollution, we assume the relative risk is monotonically increasing with exposure. To regularize the splines, and capture biologically plausible limits, we also allow a maximal derivative constraint, which is similar to penalizing total variation or limiting the spline degree. To introduce each of these constraints, we used the fact that derivatives of splines are linear functions of spline coefficients, similar to equation (1).

##### Monotonicity

We imposed monotonicity constraints using several linear inequality constraints based on exemplar exposures. Given an exemplar exposure *x*_*i*_, the requirement that the slope of the spline at exposure *x*_*i*_, be non-negative can be formulated as

$$\left\langle {X_{ij},\beta } \right\rangle \ge 0$$

for a computed vector *X*_*i*_. Linear inequality constraints were strictly enforced by the optimization solver used to fit the model, see Zheng et al.^29^.

#### Robust trimming strategy

To make the estimation of the overall relationship insensitive to potential outlying studies or observations within studies, we applied a robust, likelihood-based statistical approach—least trimmed squares (LTS)^34^—to our mixed-effects models^29^. The goal of robust statistical methods is to ensure that estimates are robust to outlying observations. Trimming approaches form a subclass of robust statistical methods, and LTS was originally developed in the context of linear regression^35^. LTS works by classifying observations into a majority of inliers and minority of outliers while simultaneously fitting the model with respect to which the inlier/outlier classification is made. Compared with other robust approaches, such as M-estimators^36^, trimming methods are more effective in limiting influence than outliers, and have a high breakdown point^37^, that is, the proportion of the data that can be arbitrarily corrupted before the estimator becomes invalid.

Trimming estimators have been applied to a broad range of problems, from linear regression^34^ to high-dimensional sparse regression and general machine learning problems^38^. In the context of mixed-effects models, trimming methods are far and away the most effective robust tools currently available for meta-analysis^29^. In practice, the approach requires only a specified inlier proportion, which was set to 90% across all examples, that is, we fit the 90% most self-coherent data points.

Using this approach, we trimmed 10% of the observations as part of the model fitting process, simultaneously discovering and fitting the most self-coherent 90% of the observations^29^. Numerical studies in data-rich cases have shown that quality of estimation is unaffected by trimming, even when there are no outliers in the data^38^. In the meta-analytic regime, the 90% level is a heuristic that balances the sparsity of available data with the need to improve estimates in the presence of outliers. This step also substantially decreased the number of risk–outcome pairs with evidence of residual publication or reporting bias.

#### Spline ensemble

To make the risk function estimates robust to knot placement, we created 50 models based on random knot placement samples. Spline estimates depend on the choice of spline parameters, including spline degree, number of knots and knot placement. To mitigate the effect of spline parameter selection on results, we developed an ensemble approach over knot placement, so that the modeler only had to specify the spline degree and number of knots.

Given the degree and number of knots, we automatically sampled a set of knot placements for a feasible knot distribution. For each knot placement, we fit a spline (including nonlinear measurements, shape constraints and trimming), evaluated each resulting model by computing its fit and curvature, and aggregated the final model as a weighted combination of the ensemble.

##### Sampling knots from simplex

We prefixed a minimal set of rules that describe a feasible set from which to sample knots, and uniformly sample from this set. Given a number of knots, the rules specify feasible ranges for each knot and feasible gaps between knots. Given an interval $\left[ {t_0,t_k} \right]$ delimited by terminal knots (which are always the minimum and maximum of the data), the feasible region of the interior knots $t_1, \ldots ,t_{k - 1}$ is denoted by

$$t_i \in \left[ {a_i,b_i} \right],\quad {\mathrm{for}}\,i = 1, \ldots ,k - 1,\quad t_i - t_{i - 1} \in \left[ {c_i,d_i} \right]\quad {\mathrm{for}}\quad i = 1, \ldots ,k.$$

We enforced the rules

$$a_i \ge t_0,\,b_i \le t_k,\quad c_i \ge 0,\quad {\sum} {c_i \le t_k - t_0.}$$

The set of knot placements that satisfy these four rules form a closed polyhedron (a volume in high-dimensional space delineated by hyperplanes). We calculated the vertices of the polyhedron using the double description method in ref. ^39^, and uniformly sampled knot placements from within the polyhedron. Each knot placement yielded a model, fit using the trimmed constrained spline approach.

##### Ensemble performance evaluation

Once the ensemble was created, we scored the resulting risk curves using two criteria: model fit (measured using the log-likelihood) and total variation (measured using the highest order derivative). These scores balanced competing objectives of fit and generalizability. Once we had these scores, denoted as *s*_1_ and *s*_2_, we normalized them to the range [0,1]:

$$v_i = \frac{{s_i - {{{\mathrm{min}}}}\left( {s_i} \right)}}{{\max \left( {s_i} \right) - {{{\mathrm{min}}}}\left( {s_i} \right)}}$$

and applied a logistic transformation. The transformation was used to make the scoring meaningful even in the presence of spurious curves in a large ensemble. We then multiplied the scores to down-weight models that are low under either criterion (fit or total variation). The final weights are normalized to sum to 1.

Using a weighted combination of these metrics, we weighted the 50 models to create the ensemble model.

#### New nonlinear covariates

For risk–outcome pairs with nonlinear relationships, we evaluated exposure levels, since this information matters for non-log-linear pairs. To do this, we took advantage of the spline model and directly captured the typical data-generating mechanism. Specifically, we used the final model that we had estimated using the robust spline ensemble to generate a nonlinear dose–response curve, which we encoded into new nonlinear ‘signal’ covariates that were later used to enable linear mixed-effects analyses. Once the nonlinear estimation was complete, the log relative risk for each data point was a function of four parameters:

$$F\left( {a_{ij},b_{ij},c_{ij},d_{ij}} \right) = \frac{{\frac{1}{{d_{ij} - c_{ij}}}\mathop {\smallint }\nolimits_{c_{ij}}^{d_{ij}} \hat f\left( x \right){\mathrm{d}}x}}{{\frac{1}{{b_{ij} - a_{ij}}}\mathop {\smallint }\nolimits_{a_{ij}}^{b_{ij}} \hat f\left( x \right){\mathrm{d}}x}}$$

where $\hat f$ is the nonlinear function obtained by estimating spline coefficients $\hat \beta$, see equation (4), $\left[ {a_{ij},b_{ij}} \right]$ delineates the reference group exposure interval and $\left[ {c_{ij},d_{ij}} \right]$ delineates the alternate group exposure interval.

We produced two new nonlinear covariates for fixed and random effects. The new nonlinear fixed-effects covariate, denoted signal^f^, is given by

5 $${\mathrm{signal}}_{ij}^{\mathrm{f}} = F\left( {a_{ij},b_{ij},c_{ij},d_{ij}} \right).$$

The new nonlinear random effect covariate, denoted by signal^r^, is given by

6 $${\mathrm{signal}}_{ij}^{\mathrm{r}} = F\left( {t,t,c_{ij},d_{ij}} \right),$$

where *t* denotes a fixed reference, for example, the theoretical minimum risk exposure level.

Using this innovation, we implemented further stages of analysis using linear mixed-effects modeling. In particular, at the end of the nonlinear stage we fit the linear mixed-effects model using only the new nonlinear covariates:

7 $$y_{ij} = {\mathrm{signal}}_{ij}^{\mathrm{f}}\beta _s + {\mathrm{signal}}_{ij}^{\mathrm{r}}u_i + {\it{\epsilon }}_{ij}$$

where ${\it{\epsilon }}_{ij}\approx N\left( {0,\sigma _{ij}^2} \right)$ are known by each observation, *β*_*s*_ is a scalar linear covariate multiplier on the signal^f^ covariate, and *u*_*i*_ is a random study-specific slope on the signal^r^ covariate with unknown variance *γ*. The posterior for *β*_*s*_ in equation (7) was used as a reference for the prior in bias covariate selection, described in step 3 of the Methods.

The relative risk between two exposure groups is a ratio of integrals of the spline across two specified intervals, so we used this exact nonlinear mechanism to inform the fit^29^. Data from studies usually compare outcome rates in one exposure alternative group to those in a separate reference group. For example, in diet cohort studies, it is common to compare the highest quartile of exposure to the lowest quartile of exposure. In trials of anti-hypertensives, the comparison of outcome rates is between the level of blood pressure in the intervention group and the level in the control group. Cohort studies of BMI often report rates in one range of BMI to a variety of reference groups, such as 20–21, 20–25 or 23–25.

Finally, for our visualizations (Figs. 1a–4a), we plotted each data point with *x* value at the midpoint exposure of the alternative group, and *y* value corresponding to the sum of the log relative risk and estimated curve evaluated at the midpoint of the reference group. These visualizations allow the standard assessment of fit quality, with a perfect fit corresponding to the estimated nonlinear relationship passing through the data.

### Testing for bias across different study designs and characteristics

Following the approach of the GRADE criteria^40^, we quantified common sources of bias across six domains: representativeness of the study population, exposure, outcome, reverse causation, control for confounding and selection bias. In the illustrative cases presented here, these variables were quantified for each study during the study extraction phase. For the set of studies on a risk–outcome association, we tested systematic variation as a function of these risk of bias variables through meta-regression. We converted the dose–response relationship identified in step 1 into a new signal covariate, effectively linearizing the non-log-linear relationship. For each bias covariate *x* (coded as an indicator variable), we defined a corresponding interaction covariate (that is, an effect modifier):

$$y_{ij} = {\mathrm{signal}}_{ij}^{\mathrm{f}} \times \left( {\beta _s + x_{ij}^1\beta _1 + \cdots + x_{ij}^k\beta _k} \right) + {\it{\epsilon }}_{ij}$$

that modified the slope of the signal covariate. We then tested risks of bias of the effect modifiers through linear meta-regression. To be included, every bias covariate must have some studies that are the gold standard (that is, at the standard of the best studies that have been conducted) for that covariate, otherwise it is not possible to incorporate it into the regression framework. Further, in considering potential covariates, we enforced that every categorical covariate had at least two studies in each category. Since bias covariates were already study specific, we only considered the fixed-effects model in bias covariate selection.

We used a robust approach to test for bias that limited the risk of overinterpreting differences with limited numbers of studies. We used the Lasso^41,42^ approach—which augments the least squares loss typically solved in a linear regression by penalizing the sum of absolute values of the bias covariate multipliers—to obtain a ranked list of bias covariates using the following equation:

8 $$\begin{array}{l}\mathop {{\min }}\limits_\beta \mathop {\sum }\limits_{i,j} \frac{1}{{2\sigma _{ij}^2}}\left( {y_{ij} - {\mathrm{signal}}_{ij}^{\mathrm{f}}\times\left( {\beta _s + x_{ij}^1\beta _1 + \cdots + x_{ij}^k\beta _k} \right)} \right)^2\\ + \frac{1}{2}\beta ^T{{\varSigma }}^{\left\{ { - 1} \right\}}\beta + \lambda \left\| \beta \right\|_1\end{array}$$

where *β* contains specifically bias covariate multipliers, *Σ* is a diagonal matrix linked to the posterior on *β*_*s*_ from the basic linear model equation (7), and the term $\lambda \left\| \beta \right\|_1$ penalizes the sum of the absolute values, pushing the bias covariate multipliers *β* to 0, with a strength determined by *λ* (ref. ^42^).

We then selected bias covariates based on their Lasso ranking, starting with a high value of *λ*.

We then added the selected covariates to the linear meta-regression model one at a time, following the Lasso ranking. To make the selection stable in the face of sparse data, we tested for significance of covariates using a Gaussian prior that biased all bias coefficients to 0 with a strength proportional to the posterior of the main dose–response relationship. If the coefficients were significant, they stayed in the model as the process continued. We terminated the process when the last added bias covariate was no longer significant after accounting for ‘signal’ and any higher-ranked covariates already in the model. We predicted the risk function using the values of the included bias covariates that reflected the preferred level of the covariate, such as the highest level of control for confounding. Supplementary Tables 11–14 provide study-specific information on study quality by risk–outcome pair.

### Quantifying between-study heterogeneity and accounting for heterogeneity, uncertainty and small numbers of studies

Estimation of between-study heterogeneity is an important aspect of meta-analysis. It reflects the variation between studies and consistency across literature.

After the selection procedure, we fit a final linear mixed-effects model that included the signal and selected bias covariates. Division by a common referent in the typical measurement mechanism induces correlation, specifically an intercept shift in log relative risk space; we therefore used a random intercept in the mixed-effects model to account for this induced within-study correlation. To capture the between-study heterogeneity, we used a study-specific random slope with respect to the signal model so that the random effect for each study effectively scaled the nonlinear relative risk curve. Formally, we fit a linear mixed-effects model of the form

$$y_{ij} = {\mathrm{signal}}_{ij}^{\mathrm{f}} \times \left( {\beta _s + x_1\beta _1 + \cdots + x_k\beta _k} \right) + {\mathrm{signal}}_{ij}^{\mathrm{f}}u_i + {\it{\epsilon }}_{ij}$$

where ${\it{\epsilon }}_{ij}\approx N\left( {0,\sigma _{ij}^2} \right)$ are the reported observation standard errors, and *u*_*i*_ are random effects with a common unknown variance,

$$u_i\approx N\left( {0,\gamma } \right).$$

Parameters *β* and *γ* were estimated simultaneously using maximum likelihood; see Zheng et al.^29^ for more details. We used the same prior on bias covariates in this analysis as we used in equation (8), that is $\beta \approx N\left( {0,\varSigma } \right)$. For log-linear relative risks, this modeling choice reduced to the classic analysis, where the random slope with respect to exposure was equivalent to the random intercept for log-linear relative risk.

To account for the small-studies problem—that is, in the setting of small numbers of studies, between-study heterogeneity (*γ*) can easily be underestimated^43^, and in particular the estimate may be 0 when too few studies are available—we quantified the uncertainty in heterogeneity estimation^13^. This estimate allowed a quantile of the heterogeneity parameter to be used, increasing the robustness of the estimate against the small-study problem. Among several alternatives in the literature^44,45^, we used the Fisher information matrix (FIM)^44^ to estimate the uncertainty of the heterogeneity. The FIM is weakly dependent on observed data, but is sensitive to the nonlinear relationship, selected bias covariates, reported standard errors and the number of studies. The final UIs we report are composed of two components: (1) posterior uncertainty corresponding to fixed effect *β*_*s*_ and (2) 95% quantile of *γ*, which depends on the estimate of *γ* and the estimate of the variance of *γ* using the inverse of Fisher information. The sensitivity analysis shows that small sample size alone did not have a significant effect on the BPRF.

### Evaluating potential for publication or reporting bias

A significant association between mean effect and standard error may indicate potential for publication or reporting bias, or methodological differences between large and small studies, which likewise lead to biased results. Publication bias is an important issue in meta-analysis^46^, and a formal test is typically done in addition to visual inspection of the funnel plot to decrease the chances of flagging apparent bias due to chance alone. In the proposed approach, we checked whether the standard errors were significant predictors of the observations in the presence of the signal and bias covariates. To detect publication bias, we used a data-driven approach known as Egger’s Regression^47^. The approach detects if there is a significant correlation between the residuals and their standard errors. When Egger’s Regression failed to detect significant evidence of publication bias, we terminated the process. While we identified these pairs as having potential for publication or reporting bias, we followed the general literature and did not incorporate any correction to the risk function based on this finding.

### Estimating the BPRF

The combined uncertainty for the mean, estimated between-study heterogeneity, and 95th quantile of the between-study heterogeneity obtained from the FIM estimate were used to generate a BPRF. The BPRF is defined as either the 5th (for harmful risks) or 95th (for protective risks) quantile curve closest to the line of relative risk equal to 1 (the null), and can be interpreted as the smallest harmful or protective effect at each level of exposure consistent with the available evidence.

In the range of exposures defined by the 15th and 85th percentiles of exposure levels observed for each risk across available studies, the ROS is defined as the signed value of the average log BPRF. For example, a log BPRF of 0.4 for a harmful risk (where null = 0) and of –0.4 for a protective risk would both have an ROS of 0.4 because the magnitude of the log relative risk is the same. In contrast, for risk–outcome pairs with a BPRF opposite the null from the mean risk (that is, the BPRF suggests that the relationship is opposite of the expected relationship—a BPRF below 1 for a harmful risk and above 1 for a protective risk), ROS would be calculated as negative.

This definition is symmetric for harmful and protective risks since the null corresponds to a log relative risk of 0. The ROS provides a single summary of the log relative risk in the range of exposure supported by the available studies, with a higher positive ROS always corresponding to a stronger relationship and a negative ROS corresponding to the situation where the available evidence fails to reject the null. For example, ROS can be negative when between-study heterogeneity is large and the relative risk function close to 1. We tested alternative ranges of exposure such as the 10th and 90th percentiles and the 5th and 95th percentiles, and the correlation of the resulting ROS with the 15th and 85th percentiles across 180 risk–outcome pairs evaluated in GBD 2020 was 0.984 and 0.979, respectively.

All risk–outcome examples presented here reflect continuous dose–response relationships. However, the risk score concept was extended to the binary risk–outcome pairs among the full 180 pairs in the analysis. The analysis for binary pairs was simpler than for continuous pairs because relative risks are comparisons between exposed and unexposed groups. The lower envelope of the log relative risk was defined analogously to the continuous risk as the 5% quantile for the effect size that included effect size uncertainty and between-study heterogeneity obtained using the 95% quantile of *γ*. To account for the fact that continuous risk–outcome analysis averages over an exposure domain, we modified the binary ROS. In the log-linear situation under basic assumptions, the averaging process reduced the score by a factor of two compared to a binary group definition that did not account for exposure. To make the continuous and binary scores comparable, we therefore divided the binary ROS by two.

To guide policy-makers and research funders when making broader comparisons across risk–outcome relationships, we converted the ROSs into star-rating categories. In this schema, one-star risks are those for which the ROS is negative and therefore the risk–outcome pair is not significant in the BPRF framework, indicating that a conservative interpretation of the evidence fails to find a significant association. We further divided the positive ROSs into ranges 0.0–0.14, >0.14–0.41, >0.41–0.62 and greater than 0.62, and assigned each range a star rating from two (0.0–0.14) through five (>0.62). Under a conservative interpretation of the evidence, exposure to a harmful risk in the average range of exposure increases the risk of the disease outcome by less than 0% for one-star pairs, 0–15% for two-star pairs, >15–50% for three-star pairs, >50–85% for four-star pairs and greater than 85% for five-star pairs compared to no exposure. Likewise, exposure to a protective risk in the average range of exposure decreases the risk of the subsequent outcome by less than 0% for one-star pairs, 0–13% for two-star pairs, >13– 34% for three-star pairs, >34–46% for four-star pairs and greater than 46% for five-star pairs compared to no exposure.

### Model validation

#### Method comparison

We validated key aspects of the meta-regression model using detailed simulation experiments.

To check the accuracy of estimating non-log-linear dose–response relationships, uncertainty and associated BPRFs, we simulated three scenarios: (1) many studies with many data points per study (30 studies, each with 4–9 observations), (2) many studies with few data points per study (30 studies, each with 1–4 observations) and (3) few studies with few data points per study (10 studies, each with 1–4 observations). In each scenario, we simulated log-linear and non-log-linear ground truth data risk functions, with three levels of between-study heterogeneity, characterized by $\gamma \in \left\{ {0.0,0.01,0.02} \right\}$. For each of these 18 combinations, we generated 100 dataset realizations to ensure that summary metrics accounted for stochastic error, for a total of 1,800 simulations.

For exposures, we used a beta distribution, supported on $x \in \left[ {0,1} \right]$, with density proportional to

$$x^{ \alpha - 1}(1 - x)^{\beta - 1}$$

with parameters *α* = 1 and *β* = 3. Using this exposure distribution, we were more likely to sample smaller exposures, giving wider range exposures in the tail of the distribution.

For each simulation, we compared the results of the approach developed here with results obtained using existing approaches with available open-source tools: log-linear meta-analysis implemented in the metafor package^10^, a meta-analysis package for linear models; and two-stage^22^ and one-stage^23^ approaches for dose–response meta-analysis, both implemented in the dosresmeta package^24^. The metafor package assumes log-linear models. We used midpoint approximations to obtain data points and used weighted least squares to summarize data to have one measure per study, as needed by metafor. The dosresmeta two-stage approach first fits a spline model for each study, then performs a meta-analysis on the estimated coefficients. We used a quadratic polynomial (the simplest model that approximates a quadratic spline) in all examples, since the complexity is limited by the necessity of fitting a model for each study. We used standard midpoint approximations as this method does not allow range exposure integration. The dosresmeta one-stage approach pools the data and does a random spline meta-analysis. Here, we compared two types of splines, the quadratic spline and the natural cubic spline, both available to practitioners who use the tools. For the proposed approach presented in this paper, we used a quadratic spline with linear tails, the ratio model and heterogeneity estimation with uncertainty obtained from the FIM. The results were tabulated across simulations to get aggregate accuracy for both mean risk function and BPRF estimation (measured using root mean squared error).

Figure 5 previews the results corresponding to the first (data-rich) scenario, to highlight the advantages of the proposed methods for non-log-linear risks. When the relationship is log-linear, our proposed approach and the metafor package (tailored for log-linear models) show somewhat better performance than competing approaches using more complex spline models. That said, the metafor package’s advantages decreased (particularly for the BPRF estimation) with increased heterogeneity. For non-log-linear relationships, our proposed approach very substantially improves on available methods, getting uniformly better performance across all scenarios and simulated between-study heterogeneity. Modeling the risk as log-linear (necessary to apply metafor) failed to capture salient features. Competing spline-based alternatives were unable to account for range exposure data, which resulted in underestimation of the mean curve. Due to the wide ranges and data sparsity at the tail, one-stage methods struggled to control the tail behavior of the splines used to estimate the risk functions. See Extended Data Figs. 1 and 2 for detailed results from the simulation of scenario one.

For the log-linear experiments in the second scenario (30 studies, 1–4 observations each), all approaches were comparable; in this sparser setting data, controlling spline tail behavior made a larger difference than in scenario one. The metafor package was on par with the proposed approach, and both were competitive compared to the one-stage and two-stage methods. For non-log-linear pairs, the proposed approach performed substantially better than all alternatives. Modeling the risk as log-linear failed to capture salient features. Competing spline-based alternatives were unable to account for interval exposure data comparing different references and alternative groups across studies. See Extended Data Figs. 3 and4 for detailed results from the simulation of scenario two.

The findings from the third scenario (10 studies, 1–4 observations each) were in line with those from the second scenario. In this data-sparse case, splines and polynomials in one-stage and two-stage methods became more unstable. Full results for scenario three are given in Extended Data Figs. 5 and 6.

#### Heterogeneity estimation simulation

We validated the utility of the FIM quantile in correcting the well-known problem of underestimating between-study heterogeneity. We generated ten scenarios parametrized by the number of studies in the dataset, ranging across 10, 20 and up to 100. For each scenario, we generated 500 realizations, for a total of 5,000 simulations. Using the quantile obtained from the FIM reduced the bias in heterogeneity estimates. See Extended Data Fig. 7 for full results.

### Sensitivity analyses

We conducted sensitivity analyses comparing the dose–response curves obtained (1) using the fixed-effects model versus the mixed-effects model and (2) with and without trimming. For the first sensitivity analysis, we removed the random effects from the fixed-effects model in the last step of our estimation process. Results are shown in Extended Data Fig. 8. There were some differences in the estimated levels of risk, and little to no difference in the shape of the risk curve between fixed- and mixed-effects of the mean risk curve.

For the second sensitivity analysis, we compared results without trimming with those after trimming the 10% least coherent data points. The results are shown in Extended Data Fig. 9 and Supplementary Table 7. We found that trimming generally stabilized the estimation, helping to guard the results against spurious observations. Trimming also decreased the estimated between-study heterogeneity, since, by definition, trimming removes points that are least coherent with the majority of the data (as judged using the model that is fit). Without trimming, ROSs and star ratings were generally lower, and more influenced by small numbers of outlying observations.

### Statistical analysis

Analyses were carried out using R v.3.6.1, Python v.3.8 and Stata v.17. To validate key aspects of the meta-regression model used in this analysis, the following packages were used: metafor (R package available for download at https://www.jstatsoft.org/article/view/v036i03) and dosmesreta (R package available for download at https://www.jstatsoft.org/article/view/v072c01).

### Statistics and reproducibility

The study was a secondary analysis of existing data involving systematic reviews and meta-analyses. No statistical method was used to predetermine sample size. As the study did not involve primary data collection, randomization, blinding and data exclusions are not relevant to this study, and as such, no data were excluded and we performed no randomization or blinding. We have made our data and code available to foster reproducibility.

### Reporting summary

Further information on research design is available in the Nature Research Reporting Summary linked to this article.

## Online content

Any methods, additional references, Nature Research reporting summaries, source data, extended data, supplementary information, acknowledgements, peer review information; details of author contributions and competing interests; and statements of data and code availability are available at 10.1038/s41591-022-01973-2.

## Supplementary information

Supplementary Information Supplementary Tables 1–14.

Reporting Summary

## Data Availability

The findings from this study were produced using data available in the published literature. Study sources and citations for each risk–outcome pair can be downloaded using the ‘download’ button on each risk curve page at https://vizhub.healthdata.org/burden-of-proof. Study characteristics for all input data used in the analyses for the four example risk–outcome pairs are also provided in Supplementary Tables 7–10.

## Code availability

All code used for these analyses is publicly available online (https://github.com/ihmeuw-msca/burden-of-proof). This includes code for the meta-regression engine, the model specification interface, both parts of the data processing, and risk-specific custom code, as appropriate.
