# Supplementary material for: The Burden of Proof studies: assessing the evidence of risk
Source: Nat Med. 2022 Oct 10;28(10):2038–44. doi: 10.1038/s41591-022-01973-2 (PMC9556298; doi:10.1038/s41591-022-01973-2)
Supplement: Supplementary file 1 — Supplementary Tables 1–14. [file 41591_2022_1973_MOESM1_ESM.pdf]

nature portfolio

https://doi.org/10.1038/s41591-022-01973-2

Supplementary information

The Burden of Proof studies: assessing the evidence of risk

In the format provided by the authors and unedited

Supplementary Information: Supplementary tables for: “The Burden of Proof studies: assessing the evidence of risk”

Table of Contents

Supplementary Table 1: The relative risk at different levels of exposure, smoking (pack-years) and lung cancer...3

Supplementary Table 2: The relative risk at different levels of exposure, systolic blood pressure (mmHg) and ischemic heart disease ...3

Supplementary Table 3: The relative risk at different levels of exposure, vegetables (g/day) and ischemic heart disease...3

Supplementary Table 4: The relative risk at different levels of exposure, unprocessed red meat (g/day) and ischemic heart disease ...4

Supplementary Table 5: Sensitivity study results (ROS, star rating, and small study effect) with trimming and without trimming ...4

Supplementary Table 6: GATHER checklist...5

Supplementary Table 7: Study characteristics for all included studies, smoking and lung cancer ...6

Supplementary Table 8: Study characteristics for all included studies, systolic blood pressure and ischemic heart disease...18

Supplementary Table 9: Study characteristics for all included studies, vegetables and ischemic heart disease......23

Supplementary Table 10: Study characteristics for all included studies, unprocessed red meat and ischemic heart disease...26

Supplementary Table 11: Study quality for all included studies, smoking and lung cancer...27

Supplementary Table 12: Study quality for all included studies, systolic blood pressure and ischemic heart disease ...31

Supplementary Table 13: Study quality for all included studies, vegetables and ischemic heart disease ...33

Supplementary Table 14: Study quality for all included studies, unprocessed red meat and ischemic heart disease ...34

Supplementary Table 1: The relative risk at different levels of exposure, smoking (pack-years) and lung cancer

The RRs with conventional and conservative UIs reference the mean RR and 95% UI from the risk curve without between-study heterogeneity (conventional) and with between-study heterogeneity (conservative) at different levels of exposure. The conservative UIs are the reported in the main text. The lower and upper bound of the 95% UI are the 2.5th and 97.5th percentiles of the 1000 draws of the relative risk for each exposure level. RR = relative risk. UI = uncertainty interval.

Exposure (pack-years)	RR with conventional UI	RR with conservative UI
0	1 (1,1)	1 (1,1)
5	1.58 (1.53, 1.64)	1.58 (1.16, 2.16)
10	2.48 (2.31, 2.66)	2.48 (1.34, 4.58)
15	3.65 (3.30, 4.04)	3.65 (1.52, 8.80)
20	5.08 (4.48, 5.77)	5.08 (1.68, 15.33)
25	6.76 (5.82, 7.84)	6.76 (1.85, 24.72)
30	8.61 (7.28, 10.18)	8.61 (2.00, 37.12)
40	11.54 (9.54, 13.96)	11.54 (2.19, 60.71)
60	13.78 (11.24, 16.91)	13.78 (2.32, 81.85)
80	14.02 (11.41, 17.22)	14.02 (2.33, 84.22)
100	14.03 (11.42, 17.23)	14.03 (2.33, 84.30)

Supplementary Table 2: The relative risk at different levels of exposure, systolic blood pressure (mmHg) and ischemic heart disease

The RRs with conventional and conservative UIs reference the mean RR and 95% UI from the risk curve without between-study heterogeneity (conventional) and with between-study heterogeneity (conservative) at different levels of exposure. The conservative UIs are the reported in the main text. The lower and upper bound of the 95% UI are the 2.5th and 97.5th percentiles of the 1000 draws of the relative risk for each exposure level. RR = relative risk. SBP = systolic blood pressure. UI = uncertainty interval.

SBP level (mmHg)	RR with conventional UI	RR with conservative UI
100	1 (1,1)	1 (1,1)
110	1.12 (1.11, 1.12)	1.12 (1.10, 1.13)
120	1.39 (1.36, 1.41)	1.39 (1.34, 1.44)
130	1.81 (1.75, 1.87)	1.81 (1.70, 1.93)
140	2.38 (2.27, 2.50)	2.38 (2.17, 2.62)
150	3.11 (2.92, 3.31)	3.11 (2.75, 3.52)
160	3.99 (3.70, 4.30)	3.99 (3.43, 4.63)
170	4.95 (4.54, 5.41)	4.95 (4.17, 5.89)
180	5.66 (5.14, 6.22)	5.66 (4.69, 6.82)
190	6.15 (5.57, 6.79)	6.15 (5.05, 7.48)
200	6.64 (5.98, 7.36)	6.64 (5.41, 8.15)

Supplementary Table 3: The relative risk at different levels of exposure, vegetables (g/day) and ischemic heart disease

The RRs with conventional and conservative UIs reference the mean RR and 95% UI from the risk curve without between-study heterogeneity (conventional) and with between-study heterogeneity (conservative) at different levels of exposure. The conservative UIs are the reported in the main text. The lower and upper bound of the 95% UI are the 2.5th and 97.5th percentiles of the 1000 draws of the relative risk for each exposure level. g = grams. RR = relative risk. UI = uncertainty interval.

Intake level (g/day)	RR with conventional UI	RR with conservative UI
0	1 (1,1)	1 (1,1)
50	0.82 (0.76, 0.87)	0.82 (0.73, 0.91)
100	0.81 (0.75, 0.87)	0.81 (0.72, 0.90)
150	0.80 (0.74, 0.86)	0.80 (0.71, 0.90)
200	0.79 (0.74, 0.86)	0.79 (0.70, 0.90)
250	0.79 (0.72, 0.85)	0.79 (0.69, 0.89)
300	0.78 (0.71, 0.85)	0.78 (0.68, 0.89)

Supplementary Table 4: The relative risk at different levels of exposure, unprocessed red meat (g/day) and ischemic heart disease

The RRs with conventional and conservative UIs reference the mean RR and 95% UI from the risk curve without between-study heterogeneity (conventional) and with between-study heterogeneity (conservative) at different levels of exposure. The conservative UIs are the reported in the main text. The lower and upper bound of the 95% UI are the 2.5th and 97.5th percentiles of the 1000 draws of the relative risk for each exposure level. g = grams. RR = relative risk. UI = uncertainty interval.

Intake level (g/day)	RR with conventional UI	RR with conservative UI
0	1 (1,1)	1 (1,1)
25	1.03 (1,1.05)	1.03 (1.02,1.04)
50	1.09 (0.99,1.18)	1.09 (1.05,1.12)
75	1.1 (0.99,1.21)	1.1 (1.06,1.14)
100	1.12 (0.99,1.25)	1.12 (1.07,1.16)
125	1.14 (0.99,1.29)	1.14 (1.08,1.19)
150	1.16 (0.99,1.33)	1.16 (1.09,1.21)
175	1.17 (0.99,1.37)	1.17 (1.1,1.23)
200	1.19 (0.99,1.41)	1.19 (1.11,1.26)

Supplementary Table 5: Sensitivity study results (ROS, star rating, and small study effect) with trimming and without trimming

IHD = ischemic heart disease. ROS = risk-outcome score. SBP = systolic blood pressure.

	Trimming			No trimming		
Risk-outcome pair	ROS	Star rating	Small study effect	ROS	Star rating	Small study effect
Smoking - lung cancer	0.73	5	1	0.18	3	1
SBP - IHD	0.70	5	1	0.50	4	0
Vegetables - IHD	0.13	2	1	0.03	2	1
Unprocessed red meat - IHD	0.01	2	0	-0.02	1	0

Supplementary Table 6: GATHER checklist

Item #	Checklist item	Reported location
Objectives and funding		
1	Define the indicator(s), populations (including age, sex, and geographic entities), and time period(s) for which estimates were made.	Main text methods overview
2	List the funding sources for the work.	Main text acknowledgments section
Data Inputs		
For all data inputs from multiple sources that are synthesized as part of the study:		
3	Describe how the data were identified and how the data were accessed.	Main text methods step 1
4	Specify the inclusion and exclusion criteria. Identify all ad-hoc exclusions.	Main text methods step 1
5	Provide information on all included data sources and their main characteristics. For each data source used, report reference information or contact name/institution, population represented, data collection method, year(s) of data collection, sex and age range, diagnostic criteria or measurement method, and sample size, as relevant.	Supplementary Tables 7–10
6	Identify and describe any categories of input data that have potentially important biases (e.g., based on characteristics listed in item 5).	Main text methods section steps 3–5
For data inputs that contribute to the analysis but were not synthesized as part of the study:		
7	Describe and give sources for any other data inputs.	N/A
For all data inputs:		
8	Provide all data inputs in a file format from which data can be efficiently extracted (e.g., a spreadsheet rather than a PDF), including all relevant meta-data listed in item 5. For any data inputs that cannot be shared because of ethical or legal reasons, such as third-party ownership, provide a contact name or the name of the institution that retains the right to the data.	Main text Data Availability statement
Data analysis		
9	Provide a conceptual overview of the data analysis method. A diagram may be helpful.	Main text methods overview
10	Provide a detailed description of all steps of the analysis, including mathematical formulae. This description should cover, as relevant, data cleaning, data pre-processing, data adjustmalets and weighting of data sources, and mathematical or statistical model(s).	Main text methods steps 1–6
11	Describe how candidate models were evaluated and how the final model(s) were selected.	Main text methods step 7 (model validation)
12	Provide the results of an evaluation of model performance, if done, as well as the results of any relevant sensitivity analysis.	Figure 5, Extended Data Figures 1–9
13	Describe methods for calculating uncertainty of the estimates. State which sources of uncertainty were, and were not, accounted for in the uncertainty analysis.	Main text methods step 4
14	State how analytic or statistical source code used to generate estimates can be accessed.	Main text Code Availability statement
Results and Discussion		
15	Provide published estimates in a file format from which data can be efficiently extracted.	Estimates can be downloaded from https://vizhub.healthdata.org/burden-of-proof/
16	Report a quantitative measure of the uncertainty of the estimates (e.g. uncertainty intervals).	UIs are given for all findings, as relevant, including in the text, figures, and tables
17	Interpret results in light of existing evidence. If updating a previous set of estimates, describe the reasons for changes in estimates.	Main text discussion
18	Discuss limitations of the estimates. Include a discussion of any modelling assumptions or data limitations that affect interpretation of the estimates.	Main text discussion

Supplementary Table 7: Study characteristics for all included studies, smoking and lung cancer

In this presentation of the data, there is one row per study. Both = males + females, or all sexes, depending on study.

Author	Year	Study name	Population	Location	Study design	Sex	Follow-up	Age start	Age end	Exposure assessment	Endpoint	Disease ascertainment	Person-years	Events	Sample size	Outcomes	Cases	Controls	Control pool
Vena	1985		White patients admitted to Roswell Park Memorial Institute	United States	Case-control	Male		35	79	Pre-admission questionnaire	Incidence	Clinical records				Lung cancer	1002	2121	Hospital
Brownson	1987		White adults in Denver metropolitan area	Colorado, USA	Case-control	Both		30	99	Personal interview	Incidence	Disease registry				Lung denocarcinoma (ICD 163)	102	233	Disease registry
Liu	1991		Farmers in Xuanwei, China	China	Case-control	Male		18	99	Administered questionnaire	Incidence	Pathological diagnosis or clinical history				Lung cancer	56	224	Population
Potter	1991		Female from the 1985 Iowa driver's license list	Iowa, United States	Nested case-control	Female		55	69	Mailed questionnaire	Incidence	Disease registry				Lung cancer (ICD 0-162)	109	2009	Population
Becher	1991		Hospital cases and controls and population controls from residence registries in the northwest	Germany	Case-control	Both		33	90	Administered interviews	Incidence	Clinical records of histologically confirmed cases				Histologically confirmed lung cancer	194	582	Hospital, Population
Chyou	1992	Honolulu Heart Program	American male of Japanese ancestry in Oahu	Hawaii, United States	Prospective cohort	Male	22	46	65	Administered interview	Incidence	Disease surveillance of hospitals with tissue confirmation		212	8009	Lung cancer (ICD-8 162.1)			
Jockel	1992		Patients and hospital controls from seven hospitals in five German cities, and population controls from the city areas	Germany	Case-control	Both		38	87	Administered interview	Incidence	Clinical records of histologically confirmed lesions				Lung cancer	194	582	Population
Chiazze	1992		Participants in the Newark (TIMA) plant cohort	Ohio, United States	Nested case-control	Both		18	99	Administered interviews	Mortality	Death records coded by a qualified nosologist				Lung cancer	144	404	Population

Chyou	1993	Honolulu Heart Program	American male of Japanese ancestry in Oahu	Hawaii, United States	Prospective cohort	Male	22	46	65	Administered interview	Incidence	Disease surveillance		227	7733	Lung cancer			
Risch	1993		Adults in metropolitan Toronto area and St. Catharine's-Niagra Falls region of southern Ontario	Canada	Case-control	Both		30	79	Administered questionnaire	Incidence	Clinical records				Lung cancer	550	1100	Population
Brockmoller	1993		Patients from one specialized hospital in Berlin	Germany	Case-control	Both		32	84	Administered interviews	Incidence	Clinical records				Lung cancer	117	272	Hospital
Sankaranarayanan	1994		Patients, visitors, and bystanders at the Regional Cancer Center	Kerala, India	Case-control	Both		30	99	Administered interviews	Incidence	Hospital disease registry				Lung cancer	281	1488	Hospital visitors
Suzuki	1994		Hospital-based cases and controls from Rio de Janiero, Brazil	Brazil	Case-control	Both		30	89	Administered interview	Incidence	Histological confirmed cases				Lung carcinomas	123	246	Hospital
Siemiatycki	1994		Patients admitted to the hospital with lung cancer at the Graduate Institute of Medical Education and Research (PGIMER), Chandigarh."	Quebec, Canada	case-control	Male				administered interview	Incidence	Hospital records				lung cancer	146	146	Varied
Siemiatycki	1995		"Departmalet of Pulmonary Medicine of Post	Quebec, Canada	case-control	Male				administered interview	Incidence	Hospital records				lung cancer (ICD C33-C34)	370	370	Population
De Stefani	1996		Cases and controls admitted to the Instituto National de Oncologia of Montevideo	Uruguay	Case-control	Male		25	84	Administered interview	Incidence	Clinical records				Lung cancer	497	994	Hospital

Lei	1996		Adults in Guangzhou	China	Case-control	Male		18	99	Administered interviews by proxy	Mortality	Vital records				Lung cancer	792	1376	Population
Cascorbi	1996		German patients select hospitals	Germany	Case-control	Both		17	84	Self-reported	Incidence	Clinical records				Lung cancer	389	1046	Hospital
De Stefani	1996		Hospital-based cases and controls from 7 major hospitals in Montevideo, Uruguay	Uruguay	Case-control	Both		30	89	Administered interviews and questionnaires	Incidence	Clinical, radiological, and endoscopic diagnoses				Lung cancer	320	640	Hospital
Yong	1997	NHANES I Epidemiologic Followup Study	Sample of the civilian noninstitutionalized population of the United States	United States	Prospective cohort	Both	22	25	74	Administered interviews and questionnaires	Incidence	Hospital records and death certificates	191292	248	10068	Lung cancer (ICD-9 162)			
Dosemeci	1997		Non-civil servant employees	Turkey	Case-control	Male		18	99	Administrative medical records	Incidence	Administrative medical records				Lung cancer	1210	2039	Clinic
Jockel	1997		Patients and population controls from Bremale and Frankfurt/Main	Germany	Case-control	Both		33	80	Administered interview	Incidence	Clinical records of histologically or cytologically confirmed cancerous legions				Lung cancer	1004	2008	Population
Pawlega	1997		Male lung cancer patients and adults in the electoral roll	Poland	Case-control	Male		30	99	Self-administered questionnaire	Incidence	Disease registry of histologically confirmed cases				Lung cancer	176	517	Population
Barbone	1997		Adults in the province of Trieste	Italy	Case-control	Male		36	98	Structured interview	Mortality	Autopsy reports				Lung cancer	755	755	Population
Hu	1997		hospital-based cases and controls from Heilongjiang Province in China	Heilongjiang, China	Case-control	Both		18	99	Administered interviews and questionnaires	Incidence	Histological confirmed cases				Lung cancer	227	454	Hospital
Liaw	1998	12-Year Follow-Up Study	Adult residents of study townships and precincts	Taiwan	Prospective cohort	Both	12	41	99	Administered interview	Mortality	Vital records	140493	127	14397	Trachea, bronchus, and lung cancer			

Wunsch-Filho	1998		Hospital based cases and controls in Sao Paolo	Brazil	Case-control	Both		33	90	Administered questionnaire	Incidence	Clinical records of histologically or cytologically confirmed cancerous legions				Lung cancer	398	1258	Hospital
Matos	1998		Patients and residents in the Province of Buenos Aires	Argentina	Case-control	Male		30	99	Administered interviews	Incidence	Clinical records				Lung cancer	200	597	Hospital
De Stefani	1998		Hospital-based cases and controls in Montevideo that were residents of Uruguay for more than 10 years	Uruguay	Case-control	Male		30	89	Administered interviews	Incidence	Clinical records				Adenocarcinoma of the lung	426	845	Hospital
Kreuzer	1998		Cases and population controls from several regions in East and West Germany	Germany	Case-control	Both		18	69	Administered questionnaire	Incidence	Histologically or cytologically confirmed cases				Lung cancer	2260	4579	Population
Nordlund	1999	Swedish prospective cohort	Adults in the Swedish population register	Sweden	Prospective cohort	Both	26	18	69	Administered interviews and questionnaires	Incidence	Disease registry and vital records		345	41710	Lung cancer			
Band	1999		Male cancer patients	Canada	Nested case-control	Male		20	99	Self-administered questionnaire	Incidence	Disease registry				Lung cancer (ICD-9 162)	2998	7265	Disease registry
Armadans	1999		Hospital-based cases and controls from a public teaching hospital in Barcelona, Spain	Spain	Case-control	Male		30	79	Administered interviews and questionnaires	Incidence	Histologically or cytologically confirmed cases				Lung cancer	325	650	Hospital
Dikshit	2000		Males in Bhopal	India	Case-control	Male		18	99	Administered questionnaire	Incidence	Disease registry				Lung cancer	163	423	Population

Mao	2001		Adults in the provinces of British Columnia, Alberta, Saskatchewan, Manitoba, Ontario, Prince Edward Island, Nova Scotia, and Newfoundland	Canada	Case-control	Both		20	99	Mailed questionnaire and phone interviews	Incidence	Disease surveillance system of hystologically confirmed cases				Lung cancer	3280	8353	Population
Simonato	2001		Hospital-based cases and controls and population-based controls in 6 countries	Sweden, Germany, United Kingdom, France, Spain, Italy	Pooled case-control	Both		15	99	Administered interviews	Incidence	Clinical records				Lung cancer	6035	14002	Varied
Rachtan	2001		Hospital-based cases and controls at a hospital in Cracow, Poland	Poland	Case-control	Female		18	99	Administered interviews and questionnaires	Incidence	Histologically confirmed cases				Lung cancer (ICD-9 162)	242	594	Hospital contacts
Kubik	2002		Hospital-based cases and controls from Prague University	Czechia	Case-control	Female		25	89	Administered interviews and questionnaires	Incidence	Microscopically verified cases				Lung cancer	269	1348	Case contacts
Ando	2003	Japan Collaborative Cohort (JACC)	Adults in 45 study areas	Japan	Prospective cohort	Both	9	40	79	Questionnaire	Mortality	Vital records	816614	597	100736	Lung cancer			
Chan-Yeung	2003		lung cancer patients from the Aichi Cancer Center, National Nagoya Hospital, and Nagoya First Red Cross Hospital	Hong Kong, China	case-control	Both		25	90	self-administed questionnaire	Incidence	Histological confirmed cases				lung cancer	282	389	Varied
Kreuzer	2003		residents of north eastern poland	Germany	case-control	Female		18	75	administered interview	Incidence	Histological confirmed cases				lung cancer	187	691	Varied
Zatloukal	2003		Female's Health Initiative Studies	Prague, Czech Republic	case-control	Female		25	89	administered interview	Incidence	Histological confirmed cases				lung cancer	334	578	Hospital

Nishino	2004	Miyagi Prefectural Cancer Registry	Residents in 14 municipalities of Miyagi Prefecture	Japan	Prospective cohort	Male	7	40	64	Self-administered questionnaire	Incidence	Clinical records, radiology and pathology records, autopsy records, mass screening records, and death certificates	166804	141	22836	Lung cancer			
Marugame	2005	Three-Prefecture Cohort Study	Adults in Miyagi, Aichi, and Osaka	Japan	Prospective cohort	Both	10	40	80	Self-administered questionnaire	Mortality	Disease registry	748935	598	88153	Lung cancer			
Sreeja	2005		female patients with newly diagnosed lung cancer from Prague University Hospital	Kerela, India	case-control	Both		25	90	self-administered questionnaire	Incidence	Hospital records				lung cancer	366	1624	Hospital Visitor
Shimazu	2008	Japan Public Health Center-based Prospective study	All registered Japanese inhabitants in the ten public health center areas	Japan	Prospective cohort	Male	14	40	69	Self-administered questionnaire	Incidence	Voluntary reports from major hospitals, disease registries, and vital records	536325	561	50364	Lung cancer			
Hosseini	2009		patients at various hospitals diagnosed with lung cancer at participating hospitals	Tehran, Iran	case-control	Both		20	99	administered interview	Incidence	clinical records, confirmed with histology and cytology				Lung cancer			
Boffetta	2010		hospital and community-based participants from 6 locations in the United States	United States (California, Hawaii, Massachusetts, Michigan, New York)	Pooled case-control	Both		18	99	Self-reported	Incidence	Histological confirmed cases				Lung cancer, bronchioloalveolar carcinoma	799	16658	Varied

Naghibzadeh -Tahami	2010		"incident lung cancer cases from Cheng Ching General Hospital and Tungs' Taichung MetroHarbor Hospital in central	Iran	case-control	Both		40	80	self-administe d questionn aire	Incidenc e	Disease Registry - Cancer				Lung cancer	242	242	Hospital Visitor
Papadopoulo s	2011		Female residents in 10 of the 11 French departemalets	France	Case-control	Fem ale		18	76	Administe red questionn aire	Incidenc e	Pathology reports and clinical records				Lung cancer	648	1423	Population
Pesch	2012		Eight European and one Canadian case-control studies in SYNERGY database	Europe and Canada	Pooled case-control	Both		18	99	Administe red interview	Incidenc e	Clinical records				Lung cancer	1316 8	16008	Varied
De Matteis	2012		Cases and population controls in 216 municipalities in Lombardy	Italy	Case-control	Both		35	79	Computer -assisted interview	Incidenc e	Tissue pathology, cytology, or review of clinical records				Lung cancer	1943	4059	Population
Thun	2013		Participants from 7 US-based cohort studies	United States	Prospect ive cohort	Both	30	55	99	Administe red questionn aire	Mortalit y	Disease registry	5724508	5905	1463 295	Lung cancer			
Bae	2013	Seoul Male Cancer Cohort	Male beneficiaries of Korean Medical Insurance Corporation	Republic of Korea	Prospect ive cohort	Male	16	40	75	Self-administer ed questionn aire	Incidenc e	Disease registries	203870	123	1427 2	Lung cancer			
He	2013		Workers at a machinery factor in Xi'an	China	Prospect ive cohort	Both	35	33.5	69.8	Administe red interviews	Mortalit y	Hystopatho logical, clinical, or radiological diagnoses	22076	45	1494	Lung cancer			

Everatt	2014	Kunas-Rotterda m Intervent ion Study (KRIS) and Multifact oral Ischemic Heart Disease Preventi on Study (MIHDP S)	Adult males in Kuanas	Lithuania	Prospect ive cohort	Male	30	40	59	Administe red interview	Incidenc e	Disease registry and vital records	133642. 4	1780	6976	Lung cancer (ICD C33-34, 162)			
Freedman	2015	NIH-AARP	AARP members	United States	Prospect ive cohort	Both	11	50	71	Self-administer ed questionn aire	Incidenc e	Disease registry		1973	4521 31	Lung cancer			
Yun	2015		Governmalet employees and teachers in Korea	Republic of Korea	Prospect ive cohort	Both	12	20	99	Self-administer ed questionn aire	Incidenc e	Disease registry	1627069 2	6491	1355 891	Lung cancer			
Schwartz	2015		newly diagnosed lung cancer patients at Hong Kong's biggest oncology center	Sao Paulo, Brazil	case-control	Both		33	90	administer ed interview	Incidenc e	Histologica l confirmed cases				lung cancer (ICD C33-C34)	1208	1069	Population
Hansen	2017		Norwegians recruited from Norwegian Counties Study, 40 Years Study, and Cohort of Norway (CONOR) Study	Norway	Prospect ive cohort	Both	39	20	103	Administe red questionn aire	Incidenc e	Disease registry	1155361 1	6534	5855 83	Carcinom as of the traches, broncus, and lung (ICD-7 162)			
Lawania	2017		newly diagnosed lung cancer patients from 15 hospitals around Germany	Chandigar h, India	case-control	Both		26	90	self-administe d questionn aire	Incidenc e	Hospital records				lung cancer (ICD C33-C34)	811	912	Population

Tindle	2018	Framing ham Heart Study	Adult residents of Framingham, Massacheusett es	United States	Prospect ive cohort	Both	28 .7	18	99	Incidence	Incidenc e	Disease surveillance through medical record review, pathology reports, and laboratory reports	384506	543	8907	Lung cancer			
Viner	2019	Alberta's Tomorro w Project	adults aged 35-69 in Alberta, Canada who answered random digit dialing and had not been previously diagnosed with cancer, live in alberta for 1 year, and speak English	Alberta, Canada	Prospect ive Cohort	Both	12 .3	35	69	Self-Report Questionn aire	Incidenc e	Linkage with Alberta Cancer Registry		2370	2660 7	prostate cancer, breast cancer, endometri al cancer, colon cancer, lung cancer, leukemia, non-hodgkin lymphom a, hematolog ical cancers			
Lai	2019		lung cancer patients registered with the Kerman Cancer Registry	Taiwan, China	case-control	Both		30	80	self-administe d questionn aire	Incidenc e	Histologica l confirmed cases				lung cancer	140	280	Population
Jin	2019		histologically confirmed lung cancer patients from Queen Mary Hosptial	China	case-control	Fem ale		18	95	self-administe d questionn aire	Incidenc e	CDC-managed local cancer registries				lung cancer	331	331	Hospital
Shimatani	2020		Taiwan"	Aichi, Japan	case-control	Both		35	81	administer ed questionn aire	Incidenc e	Histologica l confirmed cases				Lung cancer	132	132	Hospital
Hawrysz	2020		JFC study	Poland	case-control	Male		45	80	self-administe d questionn aire	Incidenc e	X-ray and CT confirmed				lung cancer	68	1808	Population

Hansen	2021	NOWAC	The Central Population Register selected a random sample of female according to the year of birth from University of Tromsø–The Arctic University of Norway.	Norway	Prospective Cohort	Female	25	30	70	Self-Report Questionnaire	Incidence	Clinical records	764512	1507	142508	lung cancer			
Weber	2021	45 and up study	adults enrolled in the 45 and up study living in New South Wales, Australia	New South Wales, Australia	Prospective Cohort	Both	10	45	99	Self-Report Questionnaire	Incidence	Linkage with Cancer Registry		18475	229028	Lung, Myelodysplasia, Small intestine, Mesothelioma, Haematopoietic, Multiple Myeloma, Thyroid, Brain, Endometrium, NHL, Ovary, Melanoma, Breast (female), Prostate, Renal and Ureter, Myeloid , Leukaemia, Kidney, Colorectum, Stomach, Gallbladder and extrahepatic bile ducts, Head and Neck, Pancreas , Bladder, Unknown primary site , Oesophagus, Liver, Larynx			

Mezzoiuso	2021	FRiCAM multicenter cohort study	all female 41-76 residing in Milan from 2003 to 2006 who had a mammogram	Italy	Prospective Cohort	Female	15	41	76	Self-Report Questionnaire	Incidence	Self-report diagnosis		9487	75324	Lung cancer			
Park	2021	Korean National Cancer Center Community Cohort	adults more than 20 years old diagnosed with any type of cancer	South Korea	Prospective Cohort	Both	23	20	90	Self-Report Questionnaire	Mortality	Clinical Records	939852.65	173	8542	Lung cancer			
Jia	2021	UK Biobank	participants in the UK biobank study, adults from England, Scotland, and Wales between 40-69	United Kingdom	Prospective Cohort	Both	5.8	40	69	Self-Report Questionnaire	Incidence	linkage with hospital records		1779	308490	Lung cancer			
Huang	2021		2015) of China"	Taiwan, China	case-control	Both		20	80	administered interview	Incidence	Hospital records, diagnosed with ICD10 C33-C44				Lung cancer			
Rusmaully	2021		patients with lung cancer from Taichung Cheng Ching Hospital, Chung Shan Medical University, and Taichung Tungs' Taichung MetroHarbor Hospital in central Taiwan	France	case-control	Female		18	75	self-administered questionnaire	Incidence	diagnosis of primary cancer of the lung				lung cancer	237	474	Varied
Zhang	2022	UK Biobank	participants in the UK biobank study, adults from England, Scotland, and Wales between 40-69	United Kingdom	Prospective Cohort	Both	7.2	40	69	Self-Report Questionnaire	Incidence	Self-report diagnosis	2,454,915	1687	344107	Lung cancer			
Guo	2022	Cancer Screening Program in Urban China	"40–74 years old residents (40–69 years old between 2012 and	China	Prospective Cohort	Both	8	40	79	Self-Report Questionnaire	Incidence	Clinical Records	6,491,000	589	282,254	Lung cancer			

Huang	2022		adults with lung cancer in Tehran, Iran without suspected pulmonay metastases from a different primary tumor	Taiwan, China	case-control	Both		29	93	administered interview	Incidence	Pathologic confirmation				Lung cancer	190	380	Hospital
Tse	2022		WELCA study	Hong Kong, China	case-control	Male		35	79	self-administed questionnaire	Incidence	Histological confirmed cases				lung cancer (ICD C33-C34)	716	757	Population

Supplementary Table 8: Study characteristics for all included studies, systolic blood pressure and ischemic heart disease

In this presentation of the data, there is one row per study.

Study name	Author and year of publication	Population	Average follow-up	Age	Endpoints	Outcome definition	Intervention group	Control group
ABCD-N	Schrier RW, et al, 2002	Normotensive type 2 diabetic subjects identified from healthcare systems.	5.3 years	40 to 74	Incidence and mortality	Myocardial infarction and heart failure	Nisoldipine or enalapril	Placebo
ACCORD, Action to Control Cardiovascular Risk in Diabetes Study	ACCORD Study group, 2010	High-risk participants with type 2 diabetes.	4.7 years	40 to 79	Incidence and mortality	Myocardial infarction and coronary heart disease	Intensive therapy	Standard therapy
ACTION Trial	Poole-Wilson PA, 2004	Ambulatory patients diagnosed with angina pectoris with and without history of myocardial infarction.	6 years	35 to 99	Incidence and mortality	Myocardial infarction, angina and heart failure	Nifedipine	Placebo
Active I	Active I Investigators, 2011	Patients with atrial fibrillation and history of CVD or hypertension prior to the study.	4.1 years	75+	Incidence and mortality	Myocardial infarction and heart failure	Irbesartan 150 and 300 mg/d	Placebo
ADVANCE	Patel A et al, 2007	Patients diagnosed with type 2 diabetes mellitus at the age of 30 years or older with history of major cardiovascular disease or at least one other risk factor for cardiovascular disease.	5 years	55 to 76	Incidence and mortality	Coronary heart disease	Perindopril 2 mg and indapamide 625 mg	Placebo
CAMELOT	Nissen SE et al, 2004	Individuals requiring coronary angiography for evaluation for chest pain or percutaneous coronary intervention with normal blood pressure and without treatment and without heart failure.	2 years	30 to 79	Incidence and mortality	Myocardial infarction and angina	Amlodipine or enalapril	Placebo
CARDIO-SIS	Verdecchia P et al, 2009[69]	Patients with a systolic blood pressure of 150 mmHg or higher, receiving antihypertensive treatment for at least 12 weeks, without diabetes	2 years	55+	Incidence and mortality	Myocardial infarction and heart failure	tight control (<130 mmHg) of systolic blood pressure	usual control (<140 mm Hg) of systolic blood pressure
DIABHYCAR	Marre M et al, 2004[52]	Individuals with type 2 diabetes that had urinary albumin excretion ≥ 20 mg/l	4 years	52 to 78	Incidence and mortality	Myocardial infarction and heart failure	Ramipril	Placebo

DREAM, Diabetes Reduction Assessment with ramipril and rosiglitazone Medication	DREAM Trial Investigators, 2006[40]	People with impaired fasting plasma glucose or impaired glucose tolerance without diabetes or cardiovascular disease.	3 years	30+	Incidence and mortality	Myocardial infarction, heart failure and angina	Ramipril	Placebo
Dutch TIA	The Dutch TIA Trial Study Group, 1993[67]	Patients who were seen by a neurologist in one of the 56 collaborating centers that had a TIA or nondisabling ischemic stroke.	2.7 years	18+	Incidence and mortality	Coronary heart disease	Atenolol	Placebo
EUROPA, European trial on Reduction of cardiac events with Perindopril in patients with stable coronary artery disease study	Fox et al, 2003[41]	Patients with evidence of coronary heart disease and without heart failure	4.2 years	45 to 75	Incidence	Myocardial infarction	Perindopril	Placebo
EWPHE, European Working Party on High Blood Pressure in the Elderly	Amery et al, 1985[34]	Patients with systolic blood pressure within the limits 160-239 mmHg without CVD.	4.6 years	60+	Mortality	Coronary heart disease	Hydrochlorothiazide + triamterene	Placebo
FEVER Felodipine Event Reduction Study	Liu L et al, 2005[48]	Individuals with systolic blood pressure of 210 mmHg or less and diastolic blood pressure less than 115 mmHg if under antihypertensive treatment; or systolic blood pressure between 160 and 210 mmHg or diastolic blood pressure between 95 and 115 mmHg if untreated.	3.3 years	50 to 79	Incidence and mortality	Coronary heart disease	Felodipine	Placebo
HOPE-3, Heart Outcomes Prevention Evaluation study 3	Lonn et al, 2016[49]	Individuals without cardiovascular disease and with at least one of the following cardiovascular risk factors: elevated waist-to-hip ratio, history of low concentration of high-density lipoprotein cholesterol, current or recent tobacco use, dysglycemia, family history of premature coronary disease, and mild renal dysfunction;	5.6 years	55+	Incidence and mortality	Myocardial infarction, heart failure and angina and revascularization	Candesartan plus hydrochlorothiazide	Placebo

HOPE, Heart Outcomes Prevention Evaluation study	Heart Outcomes Prevention Evaluation Study Investigators, 2000[44]	Individuals with history of cardiovascular disease and/or diabetes plus at least one other cardiovascular risk factor (hypertension, elevated cholesterol levels, cigarette smoking, or microalbuminuria)	5.6 years	55+	Incidence and mortality	Myocardial infarction	Ramipril 2.5 mg	Placebo
HOT, Hypertension Optimal Treatment	Hannson et al, 1998[43]	Patients with hypertension and diastolic blood pressure between 100 and 115 mmHg	3.8 years	50 to 80	Incidence and mortality	Myocardial infarction	Diastolic control target less than 80 mmHg	Placebo, diastolic control target less than 90 mmHg
HYVET	Beckett et al, 2008[36]	Population with systolic blood pressure of 160 mmHg or more.	1.8 years	80+	Incidence and mortality	Myocardial infarction and heart failure	Indapamide 1.5 mg	Placebo
MRC 2 Medical Research Council trial of treatment of hypertension	MRC Working Party,1992[53]	Hypertensive older patients without history of myocardial infarction or stroke, diabetes, impaired renal function, within the preceding three months, had impaired renal function, asthma or any serious intercurrent disease.	5.8 years	65 to 74	Incidence and mortality	Coronary heart disease	Diuretic or beta-blocker (atenolol 50 mg/d; hydrochlorothiazide 25 mg/d or 50 mg/d plus amiloride 2 5 mg/d or 5 mg/d)	Placebo
MRFIT, Multiple Risk Factor Intervention Trial*	Stamler J et al, 1989*[30]	Men who had no history of hospitalization for heart attack.	6 years	35 to 57	Mortality	Coronary heart disease	NA*	NA*
NAVIGATOR	NAVIGATOR Study Group, 2010[54]	Patients with impaired glucose tolerance, and one or more CVD risk factors or known CV disease	6.5 years	53 to 74	Incidence and mortality	Myocardial infarction, unstable angina and heart failure	Valsartan	Placebo
PART 2 The Prevention of Atherosclerosis with Ramipril trial	MacMahon S et al, 2000[51]	Patients with hospital diagnosis (within five years of enrollment) or cardiovascular disease	4.7 years	49 to 75	Incidence and mortality	Coronary heart disease, myocardial infarction and unstable angina	Ramipril	Placebo
PATS Post-stroke Antihypertensive Treatment Study	Liu L et al, 2009[47]	Individuals with a history of stroke or transient ischemic attack	2 years	47 to 73	Incidence and mortality	Myocardial infarction	indapamide 2.5 mg daily	Placebo
PEACE, Prevention of Events with Angiotensin Converting Enzyme Inhibition Trial	Braunwald et al, 2004[37]	Patients with stable coronary artery disease and normal or slightly reduced left ventricular function.	4.8 years	52 to 76	Incidence	Myocardial infarction	Trandolapril 4 mg/d	Placebo
PHARAO	Lüders S et al, 2008[50]	Internists and general practitioners with high-normal blood pressure.	3 years	50 to 85	Incidence and mortality	Myocardial infarction	Ramipril 1.5 mg	Placebo

PREVEND IT	Asselbergs et al, 2004[35]	Patients with angiographic evidence of coronary artery disease.	3 years	30 to 80	Incidence and mortality	Myocardial infarction and angina	Fosinopril 20 mg	Placebo
PREVENT	Pitt B et al, 2000[59]	Patients who had angiographic evidence of coronary artery disease.	3 years	30 to 80	Incidence and mortality	Myocardial infarction and angina	Amlodipine	Placebo
PRoFESS Prevention Regimen for Effectively Avoiding Second Strokes Study	Yusuf S, et al, 2008[70]	Patients who had had an ischemic stroke less than 90 days before randomization and whose condition was stable.	3 years	55+	Incidence and mortality	Myocardial infarction	Telmisartan	Placebo
PROGRESS The perindopril protection against recurrent stroke study	PROGRESS Collaborative Group, 2001[61]	Individuals with a history of stroke or transient ischemic attack.	3.9 years	49 to 79	Incidence and mortality	Coronary heart disease	Perindopril 4 mg	Placebo
PSC, Prospective Studies Collaboration*	Lewington et al, 2002*[31]	Adults with no previous vascular disease recorded at baseline.		40 to 89	Mortality	Ischemic heart disease	NA	NA
RENAAL	Brenner et al, 2001[38]	Patients with type 2 diabetes and nephropathy.	3.4 years	31 to 70	Incidence and mortality	Myocardial infarction and heart failure	Losartan	Placebo
SCOPE, Study on Cognition and Prognosis in the Elderly	Lithell et al, 2003[45]	Patients with mild to moderate hypertension.	3.7 years	70 to 80	Incidence and mortality	Myocardial infarction	candesartan 16 mg daily	Placebo
SHEP Systolic Hypertension in the Elderly Program	SHEP Cooperative Research Group, 1984[63]	Older population with isolated systolic hypertension.	4.5 years	60+	Incidence and mortality	Coronary heart disease	For step 1 of the trial, dose 1 was chlorthalidone 12.5 mg/d, or matching placebo; dose 2 was 25 mg/d. For step 2, dose 1 was atenolol 25 mg/d, or matching placebo; dose 2 was 50 mg/d.	Placebo
SPRINT	SPRINT Research Group, 2015[64]	Individuals with systolic blood pressure of 130-180 mmHg and an increased risk of CVD events.	3.3 years	50+	Incidence and mortality	Myocardial infarction	Intensive treatment	Standard treatment
SPS3 Secondary Prevention of Small Subcortical Strokes trial	SPS3 Study Group, 2013[65]	Individuals who had had a recent (within 180 days), symptomatic, MRI-confirmed lacunar stroke, and were without surgically amenable ipsilateral carotid artery stenosis or high-risk cardioembolic sources.	3.7 years	30+	Incidence and mortality	Myocardial infarction	Lower target <130 mmHg	higher target (130-149 mmHg)

STOP-Hypertension	Dahlöf et al, 1991[39]	Untreated patients with systolic blood pressure of 180 mmHg or above or diastolic pressure above 105 mmHg irrespective.	2 years	70 to 82	Incidence and mortality	Myocardial infarction	Atenolol 50 mg, hydrochlorothiazide 25 mg plus amiloride 2-5 mg, metoprolol 100 mg, or pindolol 5 mg.	Placebo
Syst-China	Liu L et al, 1998[46]	Older patients with isolated systolic hypertension and without cardiovascular disease	3 years	60+	Incidence and mortality	Coronary heart disease	itrendipine, with the possible addition of captopril, hydrochlorothiazide, or both	Placebo
The BBB Study	Hannson et al, 1994[42]	Treated hypertensive patients with diastolic blood pressure of 90-100 mmHg and without history or clinical signs of coronary heart disease.	5 years	45 to 67	Incidence and mortality	Myocardial infarction	Intensified treatment	Unchanged treatment to maintain diastolic blood pressure in the range 90-100
TOMHS	Neaton JD, et al, 1993[55]	Individuals not taking antihypertensive medication with diastolic blood pressure between 90 and 99 mmHg.	4.4 years	45 to 69	Incidence and mortality	Coronary heart disease	Nutritional-hygienic intervention + one of the following: placebo; chlorthalidone 15 mg/d; acebutolol 400 mg/d; doxazosin mesylate 1 mg/d for 1 month, then 2 mg/d; amlodipine maleate 5 mg/d; or enalapril maleate 5 mg/ d.	Placebo
TRANSCEND, Telmisartan Randomized Assessment Study	TRANSCEND Investigators, 2008[66]	ACE intolerant subjects with cardiovascular disease.	4.7 years	55+	Incidence and mortality	Myocardial infarction	telmisartan 80 mg/day	Placebo
UKPDS UK Prospective Diabetes Study (UKPDS 38)	UK Prospective Diabetes Study Group, 1999[68]	Hypertensive patients with type 2 diabetes without history of myocardial infarction in the previous year; current angina or heart failure.	8.4 years	25+	Incidence and mortality	Myocardial infarction	Angiotensin converting enzyme inhibitor to maximal doses or beta blocker to maximal doses	Avoid angiotensin converting enzyme inhibitors and beta blockers
VALISH Valsartan in Elderly Isolated Systolic Hypertension Study	Ogihara T et al, 2010[57]	Patients with isolated systolic hypertension.	3.07 years	70 to 84	Incidence and mortality	Myocardial infarction	Valsartan	Valsartan

Supplementary Table 9: Study characteristics for all included studies, vegetables and ischemic heart disease

In this presentation of the data, there is one row per study. Both = males + females, or all sexes, depending on study.

Author	Year	Study name	Population	Location	Study design	Sex	Follow-up	Age start	Age end	Exposure assessment	Endpoint	Disease ascertainment	Events	Sample size
Bhupathiraju	2013	Nurses' Health Study	US female registered nurses	United States	Prospective cohort	Female	24	30	55	FFQ	Incidence	Administrative medical records or disease registries	2582	113276
Bhupathiraju	2013	Health Professionals Follow-Up study	US male health professionals	United States	Prospective cohort	Male	22	40	75	FFQ	Incidence	Administrative medical records or disease registries	3607	113276
Dauchet	2010	PRIME study	Four WHO-MONICA centers in Belfast (UK), Lille (Northern France), Strasbourg (Eastern France) and Toulouse (Southwestern France).	France and Northern Ireland	Prospective cohort	Male	10	50	59	FFQ	Incidence & Mortality	Administrative medical records or disease registries	367	2297
Dauchet	2010	PRIME study	Four WHO-MONICA centers in Belfast (UK), Lille (Northern France), Strasbourg (Eastern France) and Toulouse (Southwestern France).	France and Northern Ireland	Prospective cohort	Male	10	50	59	FFQ	Incidence & Mortality	Administrative medical records or disease registries	367	2410
Dauchet	2010	PRIME study	Four WHO-MONICA centers in Belfast (UK), Lille (Northern France), Strasbourg (Eastern France) and Toulouse (Southwestern France).	France and Northern Ireland	Prospective cohort	Male	10	50	59	FFQ	Incidence & Mortality	Administrative medical records or disease registries	367	3353
Hansen	2010	Danish Diet, Cancer, and Health Study	All men and women aged 50 to 64 years, born in Denmark, living in the greater areas of Aarhus or Copenhagen, and with no previous cancer diagnosis in the Danish Cancer Registry were invited	Denmark	Prospective cohort	Male	7.7	50	64	FFQ	Incidence & Mortality	Administrative medical records or disease registries	820	25065
Hansen	2010	Danish Diet, Cancer, and Health Study	All men and women aged 50 to 64 years, born in Denmark, living in the greater areas of Aarhus or Copenhagen, and with no previous cancer diagnosis in the Danish Cancer Registry were invited	Denmark	Prospective cohort	Female	7.7	50	64	FFQ	Incidence & Mortality	Administrative medical records or disease registries	255	28318
Knekt	1994	Finnish Mobile Health Clinic	Adult men and women from rural, urban, and industrial communities in Finland	Finland	Prospective cohort	Female	14	30	69	FFQ	Mortality	Death certificates	244	2385
Knekt	1994	Finnish Mobile Health Clinic	Adult men and women from rural, urban, and industrial communities in Finland	Finland	Prospective cohort	Male	14	30	69	FFQ	Mortality	Death certificates	244	2748
Kobylecki	2015	CGPS and the Copenhagen City Heart Study	Iindividuals aged 20–100 y were invited randomly from the Danish Civil Registration System t	Denmark	Prospective cohort	Both	21	20	100	FFQ	Incidence	Administrative medical records or disease registries	2823	97203
Liu	2000	Womens' Health Study	Female health professionals who were without heart disease, stroke, or cancer (other than nonmelanoma skin cancer) at baseline.	United States	Prospective cohort	Female	5	40	68	FFQ	Incidence & Mortality	Self-report	126	39876
Miller	2017	PURE	613 communities in 18 low-income, middle-income, and high-income countries (HIC) in seven geographical	United Arab Emirates, Canada, Sweden,	Prospective cohort	Both	7.4	35	70	FFQ	Incidence	Physician diagnosis	2143	135335

			regions: North America and Europe, South America, the Middle East, south Asia, China, southeast Asia, and Africa.	Poland, Argentina, Chile, Malasia, Turkey, Iran, Occupied Palestinian territory, Brazil, South Africa, Columbia, China, India, Pakistan, Bangladesh, Zimbabwe										
Perez-Cornago	2021	European Prospective Investigation into Cancer and Nutrition	Men and women recruited through 23 centers in 10 European countries (Denmark, France, Germany, Greece, Italy, The Netherlands, Norway, Spain, Sweden and the UK)	Denmark, France, Germany, Greece, Italy, The Netherlands, Norway, Spain, Sweden and the UK	Prospective cohort	Female	12.6	35	70	FFQ	Incidence & Mortality	Administrative medical records or disease registries	8504	490311
Pietinen	1996	Alpha-Tocopherol, Beta-Carotene Cancer Prevention Study	Male smokers recruited from 14 geographic areas in southwestern Finland	Finland	Prospective cohort	Male	6.1	50	69	FFQ	Incidence & Mortality	Administrative medical records or disease registries	1399	29133
Rebello	2014	Singapore Chinese Health Study	Singapore citizens or permanent residents aged between 45-74 y who were residing at public housing estates	Singapore	Prospective cohort	Female	15	45	74	FFQ	Mortality	Administrative medical records or disease registries	638	29968
Rebello	2014	Singapore Chinese Health Study	Singapore citizens or permanent residents aged between 45-74 y who were residing at public housing estates	Singapore	Prospective cohort	Male	15	45	74	FFQ	Mortality	Administrative medical records or disease registries	1022	23501
Sharma	2014	MEC study	Large representative samples of five ethnic groups in the United States; Caucasian, African American, Native Hawaiian, Japanese American, and Latino.	United States	Prospective cohort	Male	8	45	75	FFQ	Mortality	Administrative medical records or disease registries	1140	72866
Sharma	2014	MEC study	Large representative samples of five ethnic groups in the United States; Caucasian, African American, Native Hawaiian, Japanese American, and Latino.	United States	Prospective cohort	Female	8	45	75	FFQ	Mortality	Administrative medical records or disease registries	811	91940
Sonestedt	2015	Malmö Diet and Cancer Study	All men born between 1923 and 1945 and women born between 1923 and 1950 that live in Malmö were invited via personal letters and advertisements in the local newspaper and public places to participate in the study	Sweden	Prospective cohort	Both	14	44	74	7-day diet history	Incidence & Mortality	Administrative medical records or disease registries	2921	26445
Stefler	2016	HAPIEE study	Middle-aged men and women, randomly selected from population/electoral registers in Krakow (Poland), Novosibirsk (Russia) and six cities of the Czech Republic	Czech Republic, Poland and Russia.	Prospective cohort	Both	7.1	43	71	FFQ	Mortality	Administrative medical records or disease registries	226	19333

Tognon	2014	MONICA	Randomly selected from the Central Person Register of citizens born in 1922, 1932, 1942 and 1952 living in eleven municipalities in the Copenhagen County.	Denmark	Prospective cohort	Both	14	30	85	7d food record	Incidence & Mortality	Administrative medical records or disease registries	161	1849
Yoshizaki	2020	Japan Public Health Center-Based Prospective Study	Residents of the Okinawa Prefecture (two PHC areas: Chubu from Cohort I and Miyako from Cohort II)	Japan	Prospective cohort	Male	13.2	40	74	FFQ	Incidence & Mortality	Administrative medical records or disease registries	147	7726
Yoshizaki	2020	Japan Public Health Center-Based Prospective Study	Residents of the Okinawa Prefecture (two PHC areas: Chubu from Cohort I and Miyako from Cohort II)	Japan	Prospective cohort	Female	13.2	40	74	FFQ	Incidence & Mortality	Administrative medical records or disease registries	32	8772
Yu	2014	Shanghai Women's Health Study (SWHS)	Participants from the Shanghai Women’s Health Study (SWHS) and the Shanghai Men’s Health Study (SMHS)	China	Prospective cohort	Female	9.8	40	70	FFQ	Incidence	Physician diagnosis	148	67211
Yu	2014	Shanghai Men's Health Study (SMHS)	Participants from the Shanghai Women’s Health Study (SWHS) and the Shanghai Men’s Health Study (SMHS)	China	Prospective cohort	Male	5.4	40	74	FFQ	Incidence	Physician diagnosis	217	55474
Zhang	2021	UK Biobank study	CVD-free participants aged 40-69y	United Kingdom	Prospective cohort	Female	11.2	40	69	FFQ	Incidence	Administrative medical records or disease registries	11161	462155

Supplementary Table 10: Study characteristics for all included studies, unprocessed red meat and ischemic heart disease

In this presentation of the data, there is one row per study. Both = males + females, or all sexes, depending on study.

Report	Study name	Population	Location	Study design	Sex	Follow-up	Age start	Age end	Exposure assessment	Endpoint	Disease ascertainment	Person-years	Events	Sample size
Al-Shaar 2020	Health Professionals Follow-Up Study	US male health professionals	United States	Prospective cohort	men	30	40	75	FFQ	Incidence	Administrative medical records or disease registries	1023872	4456	43272
Bernstein 2010	Nurses' Health Study	US female registered nurses	United States	Prospective cohort	women	26	30	55	FFQ	Incidence & mortality	Administrative medical records or disease registries	2050071	3162	84136
Fraser* 1999	Adventist Health Study	non-Hispanic white California Seventh-day Adventists and others living in Adventist households	California, United States	Prospective cohort	both	6	25+		surveillance program	Mortality	Administrative medical records or disease registries	180000	2716	34198
Haring 2014	Atherosclerosis Risk in Communities Study (ARIC)	community-based prospective cohort study of middle-aged adults from four US communities (Washington County, Md; Forsyth County, NC; Jackson, Miss; and suburbs of Minneapolis, Minn.)	United States	Prospective cohort	both	22	45	64	FFQ	Incidence & mortality	Death certificates	233688	1147	12066
Key 2019	European Prospective Investigation into Cancer and Nutrition (EPIC)	volunteers (mostly ages 25–70 years) from 23 centres in ten countries (Sweden, Denmark, Norway, The Netherlands, UK, France, Germany, Spain, Italy and Greece)	Sweden, Denmark, Norway, The Netherlands, UK, France, Germany, Spain, Italy and Greece	Prospective cohort	both	12.6	mean(sd)	52(10)	FFQ	Incidence	Administrative medical records or disease registries	5164551	7193	409885
Möller 2021	Danish National Survey on Diet and Physical Activity	Non-institutionalised Danish citizens without IHD at baseline	Denmark	Prospective cohort	both	9.8	15	75	Food diary	Incidence	Administrative medical records or disease registries	77214.51	439	8007
Nagao 2012	Japan Collaborative Cohort Study	People enrolled from 45 communities across Japan	Japan	Prospective cohort	both	18.4	40	79	FFQ	Mortality	Death certificates	820075	537	51683
Papier 2021	UK BIOBANK	UK volunteers, with no history of cancer except non-melanoma skin cancer	United Kingdom	Prospective cohort	both	8	37	73	24DR	Incidence	Administrative medical records or disease registries	3449832	13134	431229
Takata 2013	Shanghai Men's Health study	Men residing in urban areas of Shanghai	Shanghai	Prospective cohort	men	5.5	40	74	FFQ	Mortality	Administrative medical records or disease registries	334281	306	61128
Takata 2013	Shanghai Women's Health Study	Women residing in urban areas of Shanghai	Shanghai	Prospective cohort	women	11.2	40	70	FFQ	Mortality	Administrative medical records or disease registries	803265	306	73162
Whiteman 1999	OXCHECK Study	Patients registered with five urban practices around Luton and Dunstable (Bedfordshire, UK)	United Kingdom	Prospective cohort	both	9	35	64	FFQ	Mortality	Administrative medical records or disease registries	5586	94	10522

Supplementary Table 11: Study quality for all included studies, smoking and lung cancer

In this presentation of the data, there is one row per study.

Author	Year	Study design	Location	cv_adj_L0	cv_adj_L1	cv_adj_L2	cv_exposure_study	cv_older	cv_subpopulation	Quality score
Liu	1991	Case-control	China	1	0	0	1	0	1	3
Brownson	1987	Case-control	United States	1	1	0	1	0	1	4
Marugame	2005	Prospective cohort	Japan	1	0	0	1	0	1	3
Dikshit	2000	Case-control	India	1	1	0	1	0	1	4
Dosemeci	1997	Case-control	Turkey	1	0	0	1	0	1	3
Freedman	2015	Prospective cohort	United States	1	0	0	1	0	1	3
Bae	2013	Prospective cohort	Republic of Korea	1	0	0	1	0	1	3
Liaw	1998	Prospective cohort	Taiwan	1	1	0	0	0	0	2
Everatt	2014	Prospective cohort	Lithuania	1	0	0	1	0	0	2
Nordlund	1999	Prospective cohort	Sweden	1	1	0	1	0	0	3
Siemiatycki	1995	case-control	Quebec, Canada	0	0	0	1	0	1	2
Siemiatycki	1995	case-control	Quebec, Canada	1	1	1	1	0	0	4
Chyou	1992	Prospective cohort	United States	1	1	0	1	0	1	4
Potter	1991	Nested case-control	United States	1	1	1	1	1	1	6
Chyou	1993	Prospective cohort	United States	1	1	0	1	0	1	4
Pesch	2012	Pooled case-control	Europe, Canada	1	0	0	1	0	1	3
Jockel	1992	Case-control	Germany	1	1	0	1	0	1	4
Jockel	1997	Case-control	Germany	1	0	0	1	0	1	3
De Stefani	1996	Case-control	Uruguay	1	0	0	1	0	1	3
Lei	1996	Case-control	China	1	1	0	1	0	1	4
Pawlega	1997	Case-control	Poland	0	0	0	1	0	1	2
Wunsch-Filho	1998	Case-control	Brazil	1	1	0	1	0	1	4
Mao	2001	Case-control	Canada	1	0	0	1	0	1	3
Barbone	1997	Case-control	Italy	1	1	0	1	0	1	4
Matos	1998	Case-control	Argentina	1	1	0	1	0	1	4

De Stefani	1998	Case-control	Uruguay	1	0	0	1	0	1	3
Simonato	2001	Pooled case-control	Sweden, Germany, United Kingdom, France, Spain, Italy	1	0	0	1	0	0	2
Risch	1993	Case-control	Canada	1	1	0	1	0	1	4
Sankaranarayanan	1994	Case-control	India	1	1	1	1	0	1	5
Band	1999	Nested case-control	Canada	1	1	0	1	0	1	4
Becher	1991	Case-control	Germany	1	1	0	1	0	1	4
Brockmoller	1993	Case-control	Germany	1	1	1	1	0	1	5
Vena	1985	Case-control	United States	1	1	0	1	0	1	4
Cascorbi	1996	Case-control	Germany	1	1	0	1	0	1	4
Chiazze	1992	Nested case-control	United States	1	1	1	1	0	1	5
Ando	2003	Prospective cohort	Japan	1	1	0	1	0	0	3
De Matteis	2012	Case-control	Italy	1	0	0	1	0	1	3
He	2013	Prospective cohort	China	0	0	0	0	1	1	2
Nishino	2004	Prospective cohort	Japan	0	0	0	1	0	1	2
Papadopoulos	2011	Case-control	France	0	0	0	1	0	1	2
Shimazu	2008	Prospective cohort	Japan	0	0	0	1	0	1	2
Tindle	2018	Prospective cohort	United States	1	1	1	1	0	1	5
Yong	1997	Prospective cohort	United States	1	1	1	1	0	0	4
Hansen	2017	Prospective cohort	Norway	1	0	0	1	0	0	2
Boffetta	2010	Pooled case-control	United States	1	0	0	1	0	1	3
Yun	2015	Prospective cohort	Republic of Korea	0	0	0	1	0	1	2
Suzuki	1994	Case-control	Brazil	1	1	1	1	0	1	5
De Stefani	1996	Case-control	Uruguay	0	0	0	1	0	1	2
Hu	1997	Case-control	China	1	0	0	1	0	1	3
Kreuzer	1998	Case-control	Germany	1	0	0	1	1	1	4
Kreuzer	1998	Case-control	Germany	1	0	0	1	0	1	3

Armadans	1999	Case-control	Spain	1	1	0	1	0	1	4
Kubik	2002	Case-control	Czechia	1	0	0	1	0	1	3
Rachtan	2001	Case-control	Poland	1	1	0	1	0	1	4
Thun	2013	Prospective cohort	United States	1	0	0	1	0	1	3
Thun	2013	Prospective cohort	United States	1	0	0	1	0	0	2
Zatloukal	2003	case-control	Czech Republic	1	1	0	1	0	1	4
Hansen	2021	Prospective Cohort	Norway	1	0	0	0	0	0	1
Zhang	2022	Prospective Cohort	United Kingdom	0	0	0	1	0	0	1
Weber	2021	Prospective Cohort	Australia	0	0	0	1	0	0	1
Guo	2022	Prospective Cohort	China	1	1	1	1	0	0	4
Mezzoiuso	2021	Prospective Cohort	Italy	1	0	0	1	0	0	2
Hawrysz	2020	case-control	Poland	1	1	0	1	0	0	3
Huang	2021	case-control	Taiwan, China	1	1	0	1	0	0	3
Viner	2019	Prospective Cohort	Canada	0	0	0	1	0	1	2
Park	2021	Prospective Cohort	Republic of Korea	0	0	0	1	0	0	1
Jia	2021	Prospective Cohort	United Kingdom	1	1	1	1	0	0	4
Rusmaully	2021	case-control	France	1	0	0	1	0	0	2
Jin	2019	case-control	China	1	1	1	1	0	1	5
Tse	2022	case-control	China	1	1	1	1	0	1	5
Huang	2022	case-control	Taiwan, China	1	0	0	1	0	1	3
Hosseini	2009	case-control	Iran	0	0	0	1	0	1	2
Naghibzadeh-Tahami	2010	case-control	Iran	1	0	0	1	0	1	3
Shimatani	2020	case-control	Japan	1	1	1	1	0	1	5
Lai	2019	case-control	Taiwan, China	1	0	0	1	0	1	3
Schwartz	2015	case-control	Brazil	1	1	1	1	0	1	5
Schwartz	2015	case-control	Brazil	1	1	1	1	0	0	4
Kreuzer	2003	case-control	Germany	1	1	1	1	0	1	5
Sreeja	2005	case-control	India	1	1	0	1	0	0	3
Siemiatycki	1994	case-control	Quebec, Canada	1	1	1	1	0	0	4

Chan-Yeung	2003	case-control	China	1	1	1	1	0	1	5
Lawania	2017	case-control	India	1	1	1	1	1	1	6

cv_subpopulation: 0 for risk estimates are likely generalizable to the general population because the sample was based on the general population with reasonable exclusions for pre-existing disease states; 1 for risk estimates of sub-groups such as high-risk groups

cv_adj_L0, cv_adj_L1, cv_adj_L2: cascading dummy variables for adjustmalet level of the risk estimates (i.e., how many confounders are adjusted for in the regression model for the risk estimate). There are four adjustmalet levels, namely, 1.no adjustmalet, 2.only adjusting for age and sex, 3.adjusting for age and sex and <= 3 other covariates, and 4.adjusting for age and sex and > 3 other covariates. If the adjustmalet level is 1, cv_adj_L0=1, cv_adj_L1=1, cv_adj_L2=1; if the adjustmalet level is 2, cv_adj_L0=1, cv_adj_L1=1, cv_adj_L2=0; if the adjustmalet level is 3, then cv_adj_L0=1, cv_adj_L1=0, cv_adj_L2=0; if the adjustmalet level is 4, then cv_adj_L0=0, cv_adj_L1=0, cv_adj_L2=0.

cv_exposure_study: 0 for exposure measured multiple times and 1 for exposure measured only at baseline

cv_older: 0 if the population contains both young and old people; 1 if the population only contains old people above 65 years old.

Supplementary Table 12: Study quality for all included studies, systolic blood pressure and ischemic heart disease

In this presentation of the data, there is one row per study.

Author	Year	Exposure Measurement Score (multiple-0, single-1)	Exposure Assessment Score (objective-0 vs self-report-1)	Outcome Assessment Score (objective-0 vs self-report-1)	Confounders Score (age, sex, smoking, income, education-0, age, sex, smoking-1, age, sex-2)	Quality Score (best-0, worst-5)
ACCODRD Study group	2010	0	0	0	0	0
Active I Investigators	2011	0	0	0	0	0
Amery, et al.	1985	0	0	0	0	0
Asselbergs, et al.	2004	0	0	0	0	0
Beckett, et al.	2008	0	0	0	0	0
Braunwald, et al.	2004	0	0	0	0	0
Brenner, et al.	2001	0	0	0	0	0
Dahlöf, et al.	1991	0	0	0	0	0
DREAM Trial Investigators	2006	1	0	0	0	0
DREAM Trial Investigators	2006	1	0	0	0	0
Fox, et al.	2003	1	0	0	0	0
Hannson, et al.	1994	1	0	0	0	0
Hannson, et al.	1998	0	0	0	0	0
Heart Outcomes Prevention Evaluation Study Investigators	2000	0	0	0	0	0
Lewington, et al.	2002	0	0	0	1	1
Lithell, et al.	2003	0	0	0	0	0
Liu L, et al.	1998	0	0	0	0	0
Liu L, et al.	2009	0	0	0	0	0

Liu L, et al.	2005	0	0	0	1	1
Lonn et al.	2016	0	0	0	0	0
Lüders S, et al.	2008	0	0	0	0	0
MacMahon S, et al.	2000	0	0	0	0	0
Marre M, et al.	2004	1	0	0	0	0
MRC Working Party	1992	0	0	0	1	1
NAVIGATOR Study Group	2010	0	0	0	0	0
Neaton, JD, et al.	1993	0	0	0	0	0
Nissen SE, et al.	2004	0	0	0	0	0
Ogihara T, et al.	2010	0	0	0	1	1
Patel A, et al.	2007	0	0	0	0	0
Pitt B, et al.	2000	0	0	0	0	0
Poole-Wilson PA, et al.	2004	0	0	0	0	0
Poole-Wilson PA, et al.	2004	0	0	0	0	0
SHEP Cooperative Research Group	1984	0	0	0	0	0
PROGRESS Collaborative Group	2001	0	0	0	0	0
Schrier RW, et al.	2002	0	0	0	0	0
SPRINT Research Group	2015	0	0	0	0	0
SPS3 Study Group	2013	0	0	0	0	0
Stamler J, et al.	1989	0	0	0	1	1
TRANSCEND Investigators	2008	0	0	0	0	0
The Dutch TIA Trial Study Group	1993	1	0	0	0	0

Supplementary Table 13: Study quality for all included studies, vegetables and ischemic heart disease

In this presentation of the data, there is one row per study.

Author	Year	Exposure Measurement Score (multiple-0, single-1)	Exposure Assessment Score (objective-0 vs self-report-1)	Outcome Assessment Score (objective-0 vs self-report-1)	Confounders Score (age,sex,smoking, income,education-0; age,sex,smoking-1; age,sex-2)	Quality Score (best-0, worst-5)
Bhupathiraju	2013	0	1	0	2	3
Dauchet	2010	1	1	0	2	4
Hansen	2010	1	1	0	1	3
Knekt	1994	1	1	0	2	4
Kobylecki	2015	1	1	0	1	3
Liu	2000	1	1	1	2	5
Miller	2017	1	1	0	1	3
Perez-Cornago	2021	1	1	0	0	2
Pietinen	1996	1	1	0	2	4
Rebello	2014	1	1	0	2	4
Sharma	2014	1	1	0	2	4
Sonestedt	2015	1	1	0	1	3
Stefler	2016	1	1	0	1	3
Tognon	2014	1	1	0	2	4
Yoshizaki	2020	1	1	0	1	3
Yu	2014	1	1	0	0	2
Zhang	2021	1	1	0	1	3

Supplementary Table 14: Study quality for all included studies, unprocessed red meat and ischemic heart disease

In this presentation of the data, there is one row per study.

Report	Cohort name	Exposure Measurement Score (multiple prospective measurements-0, single baseline prospective measurement -1)	Exposure Assessment Score (objective-0 vs self-report-1)	Outcome Assessment Score (objective-0 vs self-report-1)	Confounders Score (age,sex,smoking, income or education-0; age,sex,smoking-1; age,sex-2)	Quality Score (best-0, worst-5)
Al-Shaar 2020	Health Professionals Follow-Up Study	0	1	0	1	2
Bernstein 2010	Nurses' Health Study	1	1	0	1	3
Fraser* 1999	Adventist Health Study	1	1	0	1	3
Haring 2014	Atherosclerosis Risk in Communities Study (ARIC)	0	1	0	1	2
Key 2019	European Prospective Investigation into Cancer and Nutrition (EPIC)	1	1	0	1	3
Möller 2021	Danish National Survey on Diet and Physical Activity	1	1	0	0	2
Nagao 2012	Japan Collaborative Cohort Study	1	1	0	2	4
Papier 2021	UK BIOBANK	1	1	0	0	2
Takata 2013	Shanghai Men's Health study	1	1	0	0	2
Takata 2013	Shanghai Women's Health Study	1	1	0	0	2
Whiteman 1999	OXCHECK Study	1	1	0	1	3
